# The ADAMTS family: from extracellular matrix proteases to orchestrators of fibrosis

**DOI:** 10.1186/s12964-026-02743-0

**Published:** 2026-02-13

**Authors:** Yang Yuan, Peng Guo, Yajuan Song, Zhou Yu, Baoqiang Song

**Affiliations:** 1 Department of Plastic Surgery, Xijing Hospital, Fourth Military Medical University, 127 Changle West Road, Xi’an, 710032 China; 2 Department of Burn and Plastic Surgery, The 990th Hospital of the Joint Logistic Support Force, Zhumadian, China

**Keywords:** ADAMTS, Fibrosis, ECM, TGF-β signaling, Fibroblast, Collagen

## Abstract

Fibrosis, a pathological process defined by excessive extracellular matrix (ECM) accumulation, contributes significantly to chronic organ failure worldwide. The ADAMTS (a disintegrin and metalloproteinase with thrombospondin motifs) family proteins are secreted, multi-domain matrix-associated zinc metalloendopeptidases, which have emerged as key regulators of fibrotic pathogenesis. While the ADAMTS proteins are well known for their ability to cleave ECM components such as collagens, proteoglycans, fibronectin, and fibrillins, their roles in fibrosis extend beyond conventional ECM modulators. Through precise proteolytic modification of these ECM substrates, ADAMTS members actively orchestrate upstream and core mechanisms driving fibrosis, notably TGF-β activation and fibroblast phenotype switching. Recent studies have uncovered tissue- and substrate-specific roles of individual ADAMTS members, highlighting their dual regulatory effects in fibrotic diseases and opening avenues for targeted therapeutic strategies. Despite promising preclinical results, translating ADAMTS-targeting therapies into clinical applications for fibrosis remains challenging due to their functional duality, substrate redundancy, and poorly characterized spatiotemporal specificity. This review comprehensively summarizes the proteolytic mechanisms of ADAMTS proteases toward ECM substrates, their multifaceted roles in fibrogenesis, and discusses their translational potential as therapeutic targets.

This review summarizes the emerging roles of ADAMTS proteases in the pathogenesis of fibrotic diseases. ADAMTS family members are key regulators of extracellular matrix remodeling, influencing critical processes such as TGF-β signaling, fibroblast activation, and collagen deposition. Their dual roles in promoting and suppressing fibrosis depending on specific substrates highlight their potential as therapeutic targets. Despite promising preclinical findings, the translation of ADAMTS-targeting therapies remains challenging due to functional redundancy and context-dependent effects. This review systematically synthesizes current knowledge on ADAMTS proteases in fibrosis, providing insights into their mechanisms and therapeutic potential.

## Introduction

Fibrosis, characterized by the excessive accumulation of extracellular matrix (ECM) in tissues, can lead to organ dysfunction, morbidity, and death [1]. Common diseases associated with fibrosis include cirrhosis, hepatitis, non-alcoholic steatohepatitis, chronic kidney disease, myocardial infarction, heart failure, diabetes, idiopathic pulmonary fibrosis, and scleroderma. Epidemiologically, the burden of fibrosis is significant, affecting 1 in 4 people globally and the annualized incidence of major fibrosis-related conditions is nearly 1 in 20. However, there are currently no therapies that can prevent or reverse fibrosis. This underscores the urgent need to decipher its underlying mechanisms and develop more effective therapeutic strategies.

The dysregulation of extracellular proteolysis is a hallmark of fibrotic remodeling. Historically, matrix metalloproteinases (MMPs) and a disintegrin and metalloproteinases (ADAMs) have been extensively studied for their roles in ECM turnover and growth factor shedding in fibrosis [2–4]. More recently, the ADAMTS (a disintegrin and metalloproteinase with thrombospondin motifs) family of secreted zinc metalloproteinases has emerged as another key regulator of ECM homeostasis and remodeling with functions that partially overlap yet are distinct from those of MMPs and ADAMs due to their unique structures and substrate preferences. These multi-domain enzymes are involved in the proteolytic processing of various ECM components, such as collagens, proteoglycans, fibronectin, and fibrillins, thereby influencing tissue structure, cell signaling, and mechanical properties [5–7]. Dysregulation of ADAMTS proteases has been implicated in numerous fibrotic disorders, affecting organs such as the heart, liver, kidney and skin [8–10]. Through their interactions with central fibrotic pathways, most notably the transforming growth factor-β (TGF-β) signaling cascade, and their effects on collagen metabolism and fibroblast activation, ADAMTS proteins play multifaceted roles in both promoting and suppressing fibrosis, depending on the context and specific substrate. This review comprehensively summarizes the current understanding of the ADAMTS family in fibrosis, highlighting their substrate specificities, functional mechanisms, and therapeutic potential, with the aim of providing insights for future research on the mechanisms of fibrosis and anti-fibrotic therapeutic strategies development.

## The ADAMTS family

**Fig. 1:**
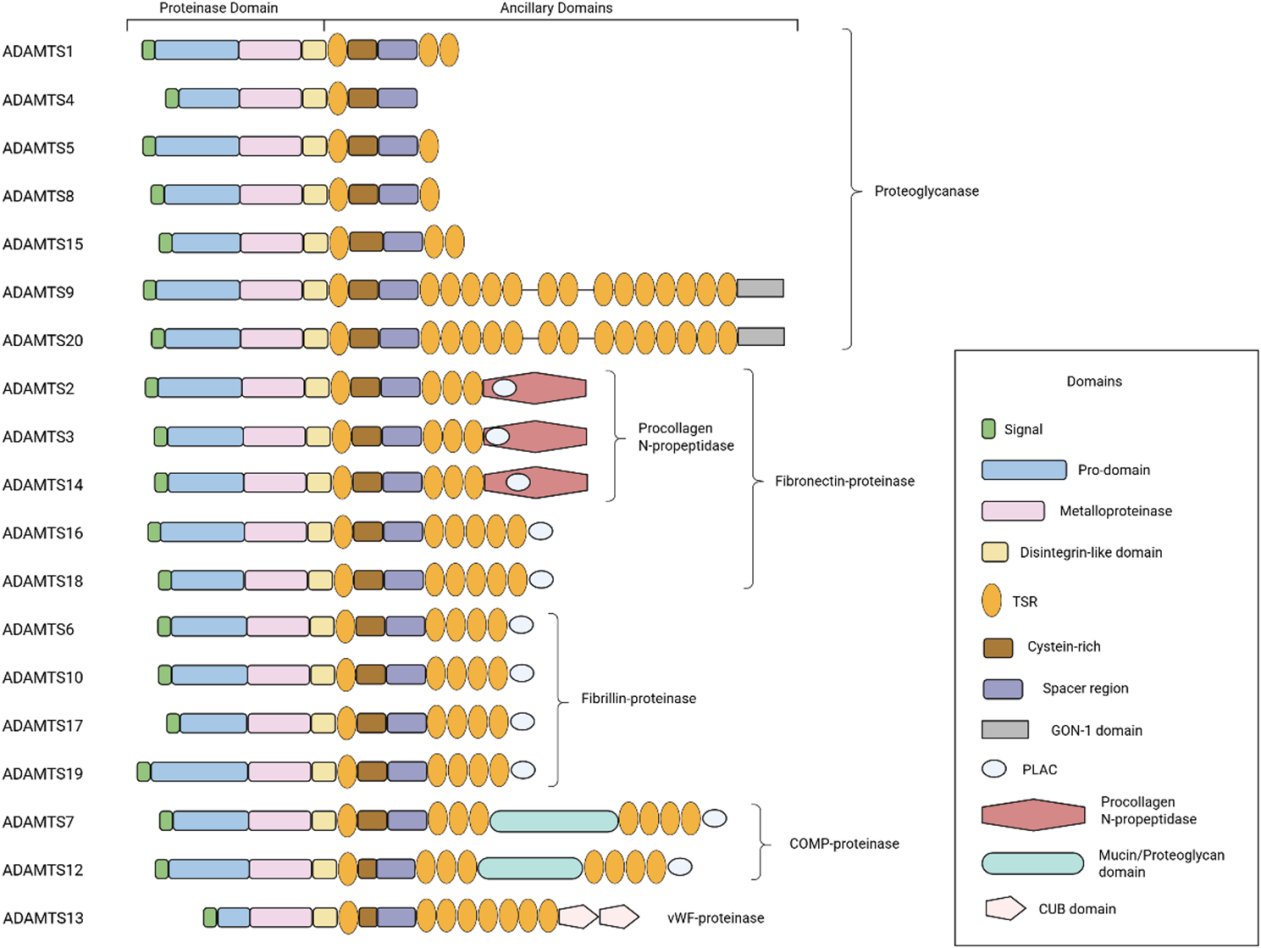
The structures of the ADAMTS family. The ADAMTS family comprises 19 members, each characterized by a conserved domain organization divided into two main regions: the proteinase domain and the ancillary domains. The proteinase domain, which is essential for enzymatic activity and substrate recognition, consists of a signal peptide, prodomain, metalloproteinase domain, and disintegrin-like domain. The ancillary domains, which exhibit the greatest variability among family members, include one or more TSRs, a cysteine-rich domain, and a spacer domain. Some ADAMTS proteases also contain additional specialized domains within their ancillary domains, further diversifying their functional roles. The overall structure is drawn to scale, highlighting the modular arrangement of these domains. COMP: Cartilage oligomeric matrix protein; vWF: von Willebrand factor; TSR: Thrombospondin type-1 repeat. This schematic was adapted from Refs [11]. The figure was prepared using BioRender (https://www.biorender.com/)

The ADAMTS enzymes are secreted, multi-domain matrix-associated zinc metalloendopeptidases that play a crucial role in ECM regulation and fibrosis processes [11]. Their structure, from the N- to C-terminus, comprises (Fig. 1): (1) A signal peptide for secretion. (2) A pro-domain that maintains proper folding and latency of the enzymes. All ADAMTS proteins contain at least one site (R/KX_n_R/K↓R) for furin-like pro-protein convertases, but the site of activation varies. Pro-ADAMTS1 and 4 are cleaved within the trans-Golgi network, pro-ADAMTS5 is processed extracellularly, and pro-ADAMTS9 is activated on the cell surface. MMPs commonly utilize a ‘cysteine switch’ controlling their activation. The dissociation of Cys73 from the zinc atom in the latent MMP and its replacement by water, with the concomitant exposure of the active site, switch the role of the zinc from a noncatalytic to a catalytic one [12]. But ADAMTSs generally lack the ‘cysteine switch’, though curiously there is evidence for this in ADAMTS15. (3) A catalytic domain that contains metalloproteinase and disintegrin-like modules. This domain features a consensus HEXXHXBG(/N/S)BXHD catalytic motif, in which the three histidines coordinate a Zn^2+^ ion [13]. Approximately 14 to 20 residues downstream of the third histidine lies a methionine, forming the ‘Met-turn’ characteristic of all metzincin catalytic domains. The metalloproteinase domain adopts the canonical metzincin fold featuring an upper N-terminal subdomain with a five-stranded β-sheet and a lower C-terminal subdomain formed by α-helices [14]. Within this helical subdomain, the Met-turn is situated, creating a hydrophobic platform beneath the catalytic Zn²⁺ shaping the active site cleft for substrate binding. The disintegrin-like domain in ADAMTS1, 4, and 5 has been characterized as a cysteine-rich region that stacks against the metalloproteinase active-site cleft and is therefore considered part of the catalytic domain [11]. To date, there is no report of any ADAMTS interacting with integrins via its disintegrin-like domain. Crystal structures of ADAMTS4 and ADAMTS5 catalytic domains reveal two conformations: an “open”, Ca²⁺-bound state and a “closed”, Ca²⁺-free state, suggesting that extracellular Ca²⁺ concentration could regulate their activity [15]. (4) Ancillary domains that mediate association with the ECM, regulation of their activity, and specification of their substrate-binding preferences. The ancillary domains of all ADAMTS proteases contain one or more thrombospondin type-1 repeats (TSRs), which are homologous to the type I repeats of thrombospondin. TSRs are believed to anchor ADAMTS proteins to the cell surface or ECM, which is associated with their interaction with glycosaminoglycans (GAGs) [16]. Additionally, TSRs of thrombospondin act as ligands for the cell surface receptor CD36, mediating the anti-angiogenic and pro-apoptotic effects on microvascular endothelial cells. This may explain why ADAMTS1, 2, 5, and 12 exert angiogenesis-blocking effects independent of their catalytic activity [17, 18]. In all ADAMTS proteases (except ADAMTS12), the first TSR is followed by a cysteine-rich region of slightly more than 100 amino acid residues including 10 conserved cysteine residues, and then, a more variable spacer region with no cysteine. Both ADAMTS9 and 20 conclude with a GON-1 module at their C-termini. They are orthologs of GON-1, an ADAMTS protease required for gonad morphogenesis in *C. elegans* [19]. ADAMTS13 is unique in having two CUB domains, which interact with its central spacer domain to autoinhibit protease activity, maintaining latency [20, 21]. The procollagen N‑peptidase domain of ADAMTS2, 3 and 14, and the mucin/proteoglycan domain of ADAMTS7 and 12, are critical for the recognition of their specific substrates.

ADAMTS enzymes are traditionally categorized by substrate and activity [11] (Fig. 1). Proteoglycanases (ADAMTS1, 4, 5, 8, 9, 15, and 20) cleave proteoglycans like aggrecan and versican via zinc-dependent proteolysis, with ancillary domains mediating ECM binding, contributing to cartilage degradation in osteoarthritis and vascular remodeling [22]. Procollagen N-propeptidases (ADAMTS2, 3, and 14) remove N-terminal propeptides from fibrillar procollagens, essential for collagen fibril assembly [23]. Mutations in ADAMTS2, 3 and 14 cause Ehlers-Danlos syndrome. vWF-cleaving protease (ADAMTS13) hydrolyzes ultra-large von Willebrand factor (vWF) regulating hemostasis; deficiency leads to thrombotic thrombocytopenic purpura [24, 25]. COMP-cleaving enzymes (ADAMTS7, 12) degrade cartilage oligomeric matrix protein (COMP), influencing vascular smooth muscle cell migration and coronary artery disease [11]. Orphan enzymes (ADAMTS6, 10, 16, 17, 18, and 19), previously characterized by the absence of well-defined substrates, are now recognized as deeply involved in microfibril assembly through interaction with fibronectin and fibrillins, linked to developmental disorders like Weill-Marchesani syndrome [26–28].

## Core substrates of ADAMTSs in ECM

The proteolytic modification of core ECM components is the defining function of the ADAMTS protease family. This section reviews the principal ECM substrates of ADAMTSs, with a focus on the detailed cleavage mechanisms and functional impacts for the major ECM components: collagens, fibronectin, proteoglycans, and fibrillins. Selected auxiliary components, including latent TGF-β binding proteins and fibulins, are also discussed, illustrating how ADAMTS proteolysis orchestrates ECM composition and dynamics.

### Collagens

**Fig. 2:**
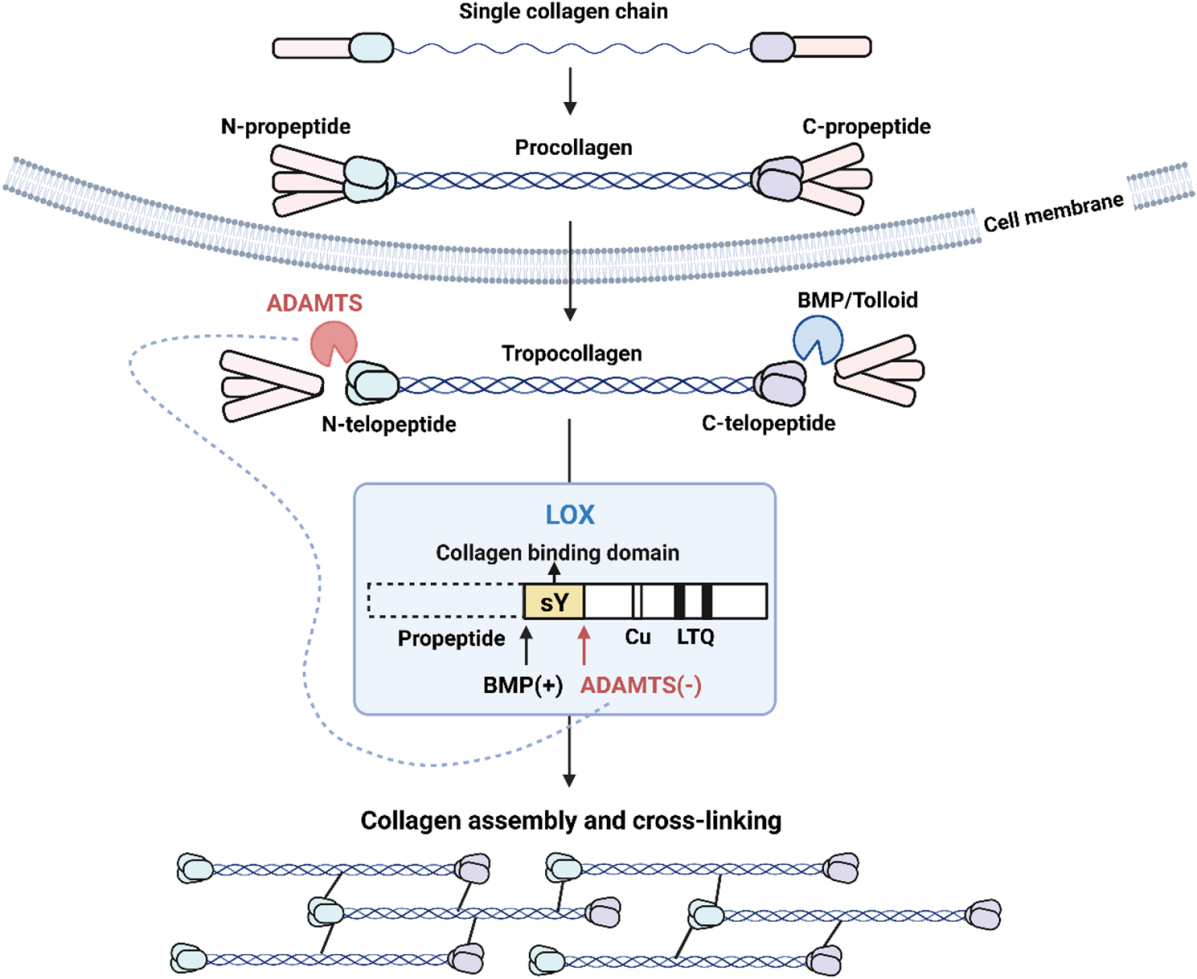
ADAMTS proteases in collagen processing and cross-linking. Various single collagen chains form procollagen, which is cleaved by ADAMTS2/3/14 (procollagen N-proteinases) and BMP/Tolloid (procollagen C-proteinases) to generate tropocollagen. LOX, featuring a collagen-binding domain, propeptide, sY, Cu, and LTQ, mediates collagen assembly and cross-linking. BMP positively regulates LOX, while ADAMTS2/14 negatively impact it. This process ensures proper collagen maturation. BMP: Bone morphogenetic protein; LOX: Lysyl oxidase. This schematic was adapted from Refs [23, 34]. The figure was prepared using BioRender (https://www.biorender.com/)

Collagens constitute 30% to 70% of ECM proteins across all tissue types and are the primary component of the ECM [29]. Fibril-forming collagens are the most abundant type of collagen and are typically found in locations that resist tensile loads, whose biosynthesis, deposition, and cross-linking are highly upregulated in fibrotic diseases. The ADAMTS proteinases, particularly ADAMTS2, ADAMTS3, and ADAMTS14, are central to the process of collagen fibril assembly and maturation (Fig. 2). Traditionally, ADAMTS2, 3, and 14 are known as procollagen N-propeptidases N-proteinases due to their procollagen N-peptide cleavage activity [23]. After synthesis, specific enzyme modification, and proper folding, fibril-forming collagen is first secreted as procollagen, which contains a large central triple-helical domain and propeptides at both ends. The amino- and carboxyl- terminal propeptides need to be cleaved to reduce the solubility of collagen and induce its almost spontaneous assembly into mature collagen fibrils. The N-terminal propeptide of procollagen is processed by ADAMTS2, ADAMTS3, and ADAMTS14, while the C-terminal propeptide is cleaved by bone morphogenetic protein(BMP)-1 and Tolloid-like family of metalloproteinases. ADAMTS-mediated cleavage of the N-terminal propeptide occurs during the process of collagen fibril assembly and is indispensable for the proper assembly of collagen fibrils [30]. In vivo, mutations in ADAMTS2 result in enzymatic dysfunction and lead to Ehlers-Danlos syndrome type VIIC, a rare connective tissue disorder caused by failed cleavage of type I procollagen [31]. When ADAMTS cleavage is impaired, procollagen retaining the N-propeptide assembles into collagen fibrils whose cross-sections are distorted from the typical circular to lobulated or thin and branched structures [32]. In addition to cleaving the procollagen N-propeptide, biochemical studies excluding BMP-1’s influence have revealed that ADAMTS2 can directly cleave the C-propeptide of type III collagen, and ADAMTS14 exhibits similar activity [33]. However, this function does not appear to be its primary role in vivo, illustrating the catalytic redundancy within the ADAMTS family.

Emerging evidence indicates that the roles of ADAMTS2 and ADAMTS14 in collagen matrix processing are more extensive than previously described [34, 35]. After cleavage of the propeptides, the non-helical telopeptide domains remain at both ends of the collagen molecue. The lysine or hydroxylysine residues within these telopeptide domains are oxidatively deaminated by lysyl oxidase (LOX) to produce the corresponding aldehydes, which eventually condense with other oxidized groups or intact lysine on collagen to form various inter- and intra- chain cross-links, greatly enhancing the mechanical strength of the collagen matrix. Rosell-García et al. have demonstrated that both ADAMTS2 and ADAMTS14 are capable of cleaving LOX and, consequently, inhibit collagen cross-linking by disrupting the interaction between the catalytic domain of LOX and collagen. This mechanism may serve to prevent procollagen from forming cross-links before its propeptides are cleaved, which would otherwise impede its secretion from the cell. Interestingly, the procollagen C-peptidases BMP-1 and Tolloids cleave the N-terminal propeptide of LOX upstream of the ADAMTS2 hydrolysis site, promoting the interaction between LOX and collagen [34]. ADAMTS and BMP-1/Tolloids collectively orchestrate a sophisticated, multi-tiered regulatory system governing collagen maturation.

### Fibronectin

Fibronectin (FN) exists in two primary forms generated through alternative splicing of a single gene: (1) soluble plasma FN, which is secreted into the circulation by hepatocytes, and (2) insoluble cellular FN, secreted by a variety of cell types including fibroblasts [36, 37]. The latter (cellular FN) resides within the ECM, where it serves as a critical template for the assembly of macromolecules such as collagens, fibrillins, latent TGF-β-binding proteins, and tenascin-C. It also functions as an essential cell adhesion molecule and a pro-migratory substrate. The primary structure of FN, from the amino- to the carboxy-terminus, consists of type I, type II, and type III repeats, followed by the alternatively spliced type III extradomains (EDA and/or EDB) [38]. The initiation of FN fibril assembly is triggered by specific interactions between FN and cell surface integrin receptors, primarily mediated through the N-terminal heparin-binding domain encompassing the first five type I repeats [39]. Only cellular FN (but not plasma FN) may contain the alternatively spliced extradomains EDA and/or EDB. EDA⁺ FN, EDB⁺ FN, and EDA⁺/EDB⁺ FN (i.e., containing both EDA and EDB) are transiently expressed during embryogenesis but are not normally present in adult connective tissues [36, 37]. The presence of EDA⁺ FN is characteristic of tissue repair and fibrosis, whereas EDB⁺ FN is most frequently associated with tumor development and angiogenesis.

**Fig. 3:**
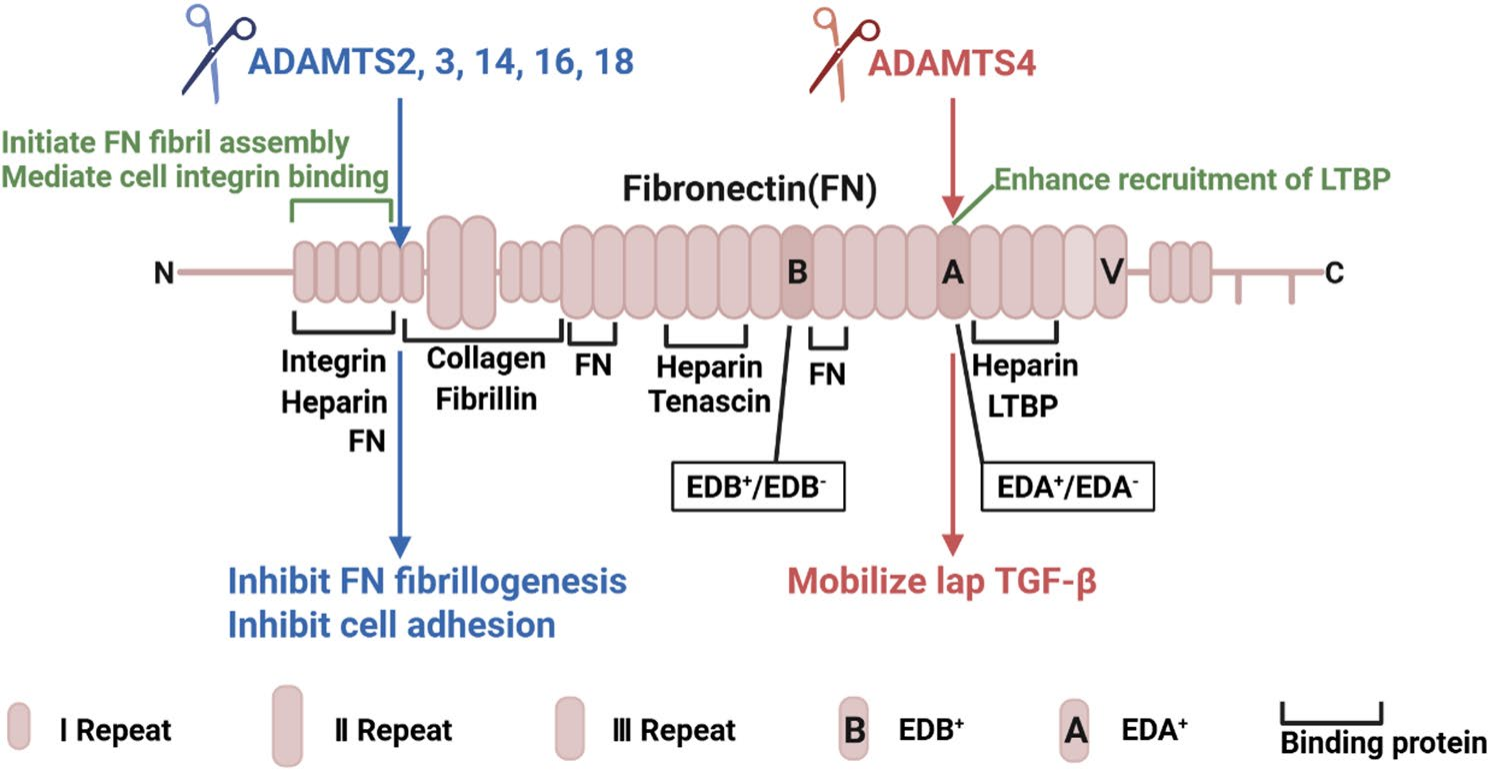
The structure of FN and ADAMTS-mediated cleavages of FN. FN, consisting of type I, type II, and type III repeats and EDA and/or EDB, serves as a scaffold for ECM components (e.g., collagens, fibrillins) and recruits LTBP. ADAMTS2/3/14/16/18 cleave FN between the fifth and sixth type I repeat and release the heparin-binding domain, inhibiting fibrillogenesis and cell adhesion. ADAMTS4 cleaves within the EDA domain, mobilizing latent TGF-β and promoting fibrotic signaling. FN: Fibronectin; EDA: Extradomain A; EDB: Extradomain B; TGF-β: Transforming growth factor-β; LTBP: Latent TGF-β binding protein. This schematic was adapted from Refs [38]. The figure was prepared using BioRender (https://www.biorender.com/)

ADAMTSs proteolytically cleave FN, influencing FN matrix assembly, its modulation of TGF-β signaling and cell adhesion (Fig. 3). Bekhouche et al. discovered that ADAMTS2, 3, and 14 can all cleave FN within the N-terminal type I repeats, generating a product of approximately 33 kDa. Furthermore, using a novel method termed amino terminal oriented mass spectrometry of substrate (ATOMS) coupled with tandem mass tag (TMT) labelling and multienzymatic digestion, they unambiguously identified the cleavage site as VRAA²⁹²↓²⁹³VYQP [33]. Schnellmann et al. developed a mass spectrometry-based approach utilizing an in vitro generated cell-free ECM [27]. They demonstrated that ADAMTS16 cleaves FN between its fifth and sixth type I repeat, releasing the N-terminal 30 kDa heparin-binding domain essential for FN self-assembly and cell adhesion [27, 37]. This proteolytic event requires a distant binding site on FN for ADAMTS16 and is likely facilitated by cellular traction forces that expose the scissile bond. Cleavage by ADAMTS16 inhibits FN assembly, consequently suppressing FN fibrillogenesis and the incorporation of other ECM components, ultimately impairing the formation of a mature ECM. Ataca et al. reported that ADAMTS18 also cleaves FN, generating a 30 kDa fragment [7]. Given its high sequence homology with ADAMTS16, ADAMTS18 is predicted to cleave FN in a mechanistically similar manner. Of particular note, Vistnes et al. observed an approximately 180 kDa FN fragment following ADAMTS4 treatment of human cardiac fibroblast lysates [40]. The absence of immunoreactivity with an EDA-specific antibody suggests proteolytic disruption within or adjacent to the EDA domain, with the fragment size further supporting cleavage in this region [40]. This pattern contrasts with the cleavage sites of ADAMTS2, 3, 14, 16, and 18, which are located outside the EDA domain [40]. Given the well-established role of EDA⁺ FN in fibrotic remodeling in cardiac and pulmonary tissues, the specific targeting of this domain by ADAMTS4 suggests significant potential for ADAMTS4 in the regulation of fibrotic pathways [41, 42].

### Proteoglycans

Versican and aggrecan are large chondroitin sulfate proteoglycans in the ECM [43]. Unlike collagens, which mediate mechanical force transmission and provide tensile resistance, versican and aggrecan primarily fill extracellular spaces and are crucial for maintaining tissue hydration and regulating cellular signaling [44]. Versican contains three principal structural domains with distinct functions: an N-terminal G1 (globular) domain that mediates hyaluronic acid binding via two link protein-like modules; one or both of two alternatively spliced, extended GAG attachment domains (GAGα and GAGβ); and a C-terminal G3 domain that interacts with diverse ECM molecules, including type I collagen, FN, fibrillins, fibulins, tenascin-R, and integrin β1 [45]. Versican is expressed as four major isoforms. All isoforms share the conserved N- and C-terminal globular domains (G1 and G3), but differ in the presence or absence of the central glycosaminoglycan attachment domains GAGα and GAGβ. Versican V1 contains only the GAGβ domain, V2 contains only the GAGα domain, V0 contains both GAGα and GAGβ domains, and V3 lacks both domains. Versican interacts with cytokine, selectin, apolipoprotein, and CD44 through its GAG side chains. For example, versican can modulate fibroblast maturation by altering intracellular signaling through interactions with hyaluronic acid and its receptor CD44 [46].

**Fig. 4:**
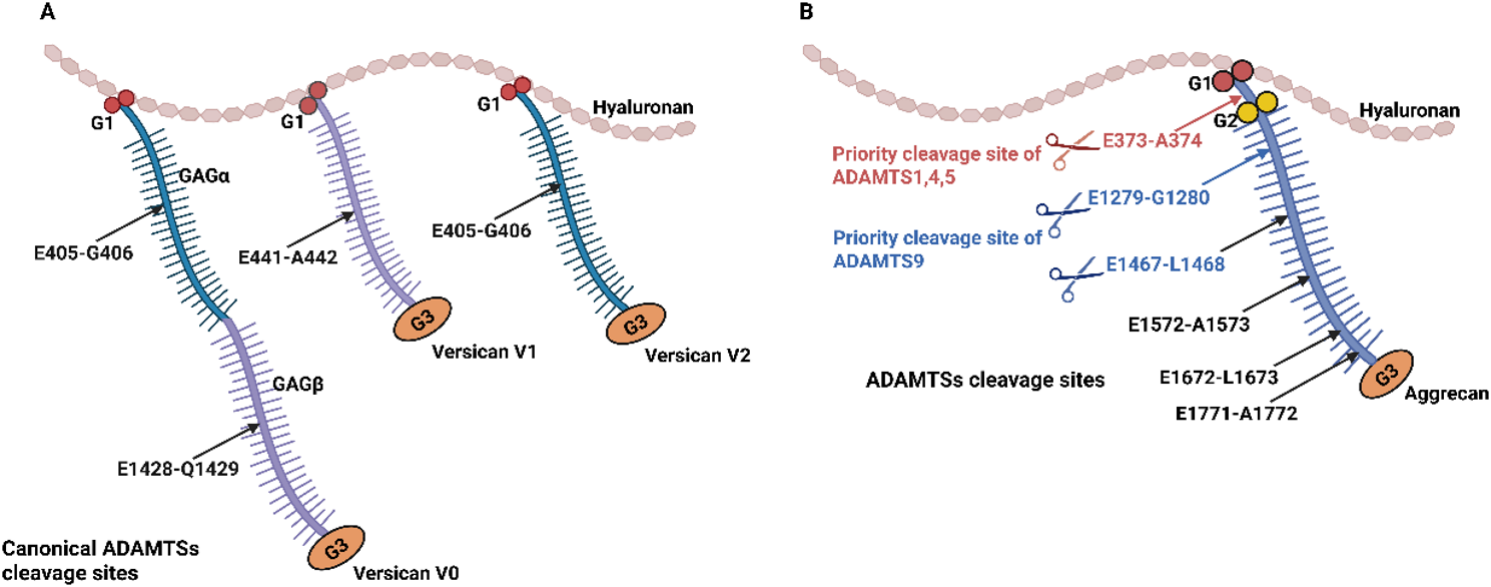
Proteolytic cleavages of versican and aggrecan by ADAMTS proteases. **A** Versican contains three principal structural domains: an N-terminal G1 domain; one or both of two alternatively spliced, extended GAG attachment domains (GAGα and GAGβ); and a C-terminal G3 domain. Versican V1 contains only the GAGβ domain, V2 contains only the GAGα domain, V0 contains both GAGα and GAGβ domains, and V3 lacks both domains. The specific cleavage sites for ADAMTS proteases are located within the GAGα and GAGβ domains of versican: one site in the V1 isoform at Glu⁴⁴¹-Ala⁴⁴² within GAGβ; one site in the V2 isoform at Glu⁴⁰⁵-Gln⁴⁰⁶ within GAGα; and two sites in the V0 isoform at Glu⁴⁰⁵-Gln⁴⁰⁶ in GAGα and Glu¹⁴²⁸-Ala¹⁴²⁹ in GAGβ. **B** Aggrecan consists of N-terminal G1 and C-terminal G3 domains flanking a central GAG domain. There are five aggrecan cleavage sites of ADAMTS proteases. ADAMTS1/4/5 preferentially cleave aggrecan at the Glu³⁷³-Ala³⁷⁴ bond between the G1 and G2 domains. ADAMTS9 preferentially cleaves aggrecan at the Glu¹²⁷⁹-Gly¹²⁸⁰ and Glu¹⁴⁶⁷-Gly¹⁴⁶⁸ bonds within the GAG domain. G: Globular; GAG: Glycosaminoglycan. This schematic was adapted from Refs [22, 47]. The figure was prepared using BioRender (https://www.biorender.com/)

ADAMTS1, 4, 5, 8, 9, 15, and 20 exhibit versican degrading activity [22, 47](Fig. 4). The canonical cleavage sites for these proteases are located within the GAGα and GAGβ domains of versican: one site in the V1 isoform at Glu⁴⁴¹-Ala⁴⁴² within GAGβ; one site in the V2 isoform at Glu⁴⁰⁵-Gln⁴⁰⁶ within GAGα; and two sites in the V0 isoform at Glu⁴⁰⁵-Gln⁴⁰⁶ in GAGα and Glu¹⁴²⁸-Ala¹⁴²⁹ in GAGβ. A recent study employed a quantitative label-free proteomic strategy to characterize versican cleavage by ADAMTS1/4/5 [48]. By quantifying the abundance of semi-tryptic peptides identified by liquid chromatography-tandem mass spectrometry (LC-MS/MS) that were generated through proteolytic processing, the study revealed multiple previously unrecognized cleavage sites within the versican V1 isoform for these enzymes [48]. The proteolytic fragment generated by cleavage at the Glu⁴⁴¹-Ala⁴⁴² bond in versican V1 is designated as versikine.

Aggrecan shares structural homology with versican, featuring conserved N-terminal G1 and C-terminal G3 domains flanking a central GAG domain. Distinctively, aggrecan contains an additional G2 domain between its G1 and GAG regions [45]. ADAMTS proteases cleave aggrecan with varying specificity at several defined sites (Fig. 4). ADAMTS1/4/5 primarily cleave aggrecan at the Glu³⁷³-Ala³⁷⁴ bond between the G1 and G2 domains [47]. Five additional ADAMTS cleavage sites have been identified within the GAG domain [22]. In comparison, ADAMTS9 preferentially targets the Glu¹²⁷⁹-Gly¹²⁸⁰ and Glu¹⁴⁶⁷-Gly¹⁴⁶⁸ bonds within the chondroitin sulfate-rich region of the GAG domain, rather than the Glu³⁷³-Ala³⁷⁴ site [49]. Unlike the proteolytic activities of other ADAMTS proteases, which are often linked to pathological conditions, cleavage by ADAMTS9 is primarily associated with the normal turnover of hyaluronic acid.

In contrast to aggrecan and versican, small leucine-rich repeat proteoglycans (SLRPs) bear far fewer GAG chains [50]. Fibrosis-associated SLRP members, such as biglycan, lumican, decorin, and fibromodulin, are often upregulated and drive pathological progression. As major structural components of the interstitial ECM in connective tissues (e.g., skin, cornea, bone, tendon, and cartilage), SLRPs interact with fibrillar collagens to regulate collagen fibril assembly [51]. Structurally, they typically consist of two parts: an N-terminal variable domain, which may contain sulfated tyrosines or acidic amino acid stretches, and a conserved C-terminal domain composed of leucine-rich repeats. All known collagen-binding sites reside within the leucine-rich repeat domain. Both ADAMTS4 and ADAMTS5 can degrade decorin, biglycan, lumican, and fibromodulin [22, 52, 53]. In human articular cartilage, ADAMTS4 cleaves fibromodulin, producing a fragment approximately 5–10 kDa smaller than the full-length protein. Moreover, ADAMTS4 recognizes a single cleavage site in the fifth leucine-rich repeat of biglycan (N^149^↓^150^C). The proteolytic activity of ADAMTS4 and ADAMTS5 against decorin, biglycan, and fibromodulin is enhanced by the sequential inclusion of their carboxyl-terminal domains [54].

### Fibrillins

Fibrillins (FBNs) are ECM glycoproteins [55]. In humans, the FBN family comprises three subtypes (FBN1, FBN2, and FBN3), whereas in mice, only two subtypes (FBN1 and FBN2) are expressed. The domain structure of the FBN consists primarily of arrays of epidermal growth factor-like (EGF) domains interspersed with TGFβ-binding-like (TB) domains and hybrid domains. FBNs undergo homotypic or heterotypic self-assembly via head-to-tail and lateral interactions to form microfibrils. Immunolocalization studies in fetal tissues showed that FBN1 either coassembles with other FBN1 molecules to form a homopolymeric microfibril or coassembles with FBN2 into a heteropolymeric microfibril [56]. Interestingly, homopolymeric interactions of FBN2 molecules were not seen. Microfibrils serve as a scaffold for tropoelastin deposition, leading to the formation of mature elastic fibers. Microfibrils also play key roles in regulating TGF-β signaling, primarily by maintaining TGF-β latency and modulating its activation, as well as sequestering BMP-1 within the ECM [57]. Structural defects in FBN, as observed in the tight-skin mouse model carrying FBN1 mutations, disrupt microfibril integrity, leading to dysregulated TGF-β signaling, myofibroblast activation, and excessive collagen deposition [58]. FBN1 mutations associated with disorders such as stiff skin syndrome impair integrin binding and trigger pro-fibrotic signaling [57]. FBN deficiency disrupts growth factor sequestration and mechanotransduction, underscoring its critical role in ECM remodeling and fibrogenesis [57].

**Fig. 5:**
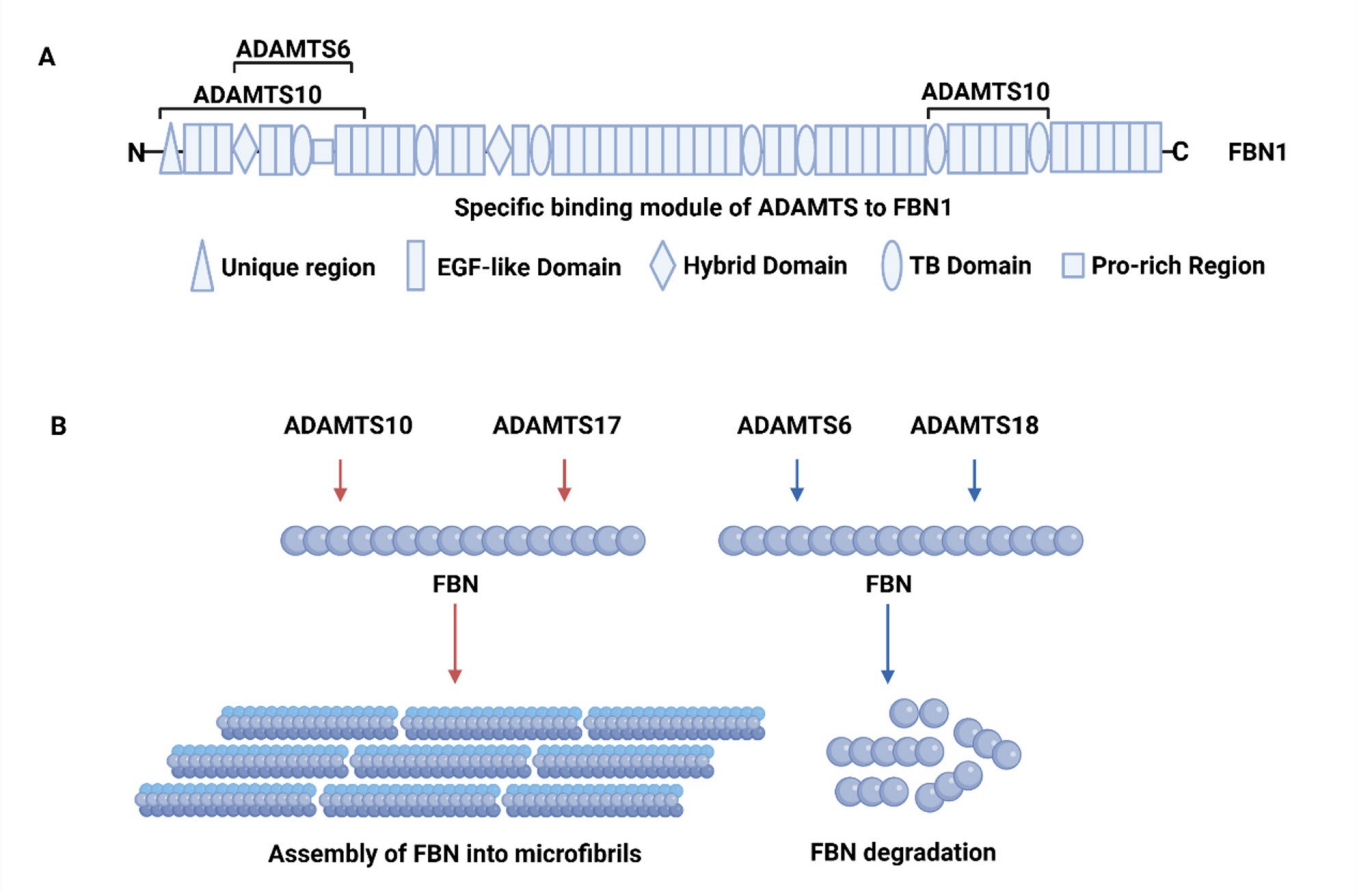
The action of ADAMTS proteases on FBN and its assembly. **A** Domain structure of FBN1 and the specific modules mediating ADAMTS binding. **B** Opposing regulatory roles of ADAMTS proteases. ADAMTS10 and ADAMTS17 bind to FBN and promote its assembly into microfibrils, whereas ADAMTS6 and ADAMTS18 bind to FBN and mediate its degradation. FBN: Fibrillin; EGF: Epidermal growth factor-like; TB: TGFβ-binding like. This schematic was adapted from Refs [55]. The figure was prepared using BioRender (https://www.biorender.com/)

Certain ADAMTS family members interact with FBN and regulate microfibril abundance [59](Fig. 5). ADAMTS10 has two FBN1 binding sites, one near the N terminus and another in the C-terminal half of FBN1 [60]. The binding site for ADAMTS10 in the FBN1 N-terminus was mapped to exons 1-11. Due to its innate resistance to furin cleavage, ADAMTS10 has low efficiency in cleaving FBN1. When recombinant wild-type ADAMTS10 (non-furin-processed ADAMTS10) is added to human dermal fibroblasts, it promotes the deposition of microfibrils. Therefore, ADAMTS10 likely plays a more prominent role in regulating the assembly of FBN1 into microfibrils than in mediating FBN1 proteolytic hydrolysis. However, a persistence of FBN2 immunoreactivity was observed in the postnatal ciliary zonule of ADAMTS10-deficient eyes, which suggests that ADAMTS10 promotes the formation of FBN1 into microfibrils while suppressing FBN2 [61]. But after furin processing is enabled to ADAMTS10, ADAMTS10 exerts proteolytic activity on both FBN1 and FBN2 in vitro [26]. ADAMTS6, the sister protease of ADAMTS10, binds to a similar N-terminal region of FBN1(exons 6-11). Overexpression of ADAMTS6 in epithelial cells did inhibit FBN1 microfibril formation, but the underlying mechanism remains unclear [62]. It is plausible that ADAMTS10 may promote microfibril formation independent of its proteolytic activity while ADAMTS6 may work as a true protease. ADAMTS18 acts similar to ADAMTS6. Lu, T. et al. found that ADAMTS18 colocalizes with FBN1 and FBN2 in the ECM of cultured fibroblasts [63]. ADAMTS18 deficiency leads to increased levels of FBN1 and FBN2 and the accumulation of microfibrils in the bronchi [63].

ADAMTS17 binds recombinant FBN2 but not FBN1 and does not cleave either [64]. Currently, the binding sites of ADAMTS17 and ADAMTS18 on FBN remain unelucidated. Interestingly, ADAMTS17 colocalizes to FBN1 containing microfibrils in cultured fibroblasts. In contrast to ADAMTS10, ADAMTS17 is furin processed and undergoes extensive autoproteolysis to activate itself resulting in the release of multiple ADAMTS17 peptides. In primary skin fibroblasts from patients with WMS due to ADAMTS17 mutations, a reduction is observed in FBN1 secretion and FBN1 deposition in the ECM [65]. This suggests that ADAMTS17 or its autoproteolytic peptides could play a role in the maturation or biogenesis of FBN microfibrils. FBN microfibrils could also bring ADAMTS10 and ADAMTS17 together allowing ADAMTS17 to activate ADAMTS10 [26].

### Other substrates

Latent TGF-β binding proteins (LTBPs) serve as critical extracellular reservoirs for TGF-β, including four subtypes. LTBP1 and LTBP3 appear to associate with all three TGFβ isoforms. LTBP4 is reported to bind only LAP-TGF-β1 and does so very inefficiently, whereas LTBP2 does not bind any TGF-β isoform [66]. Recent studies have identified several ADAMTS proteases as key regulators of LTBP processing. Among them, ADAMTS2 cleaves human LTBP1 at the L^719^↓N^720^ bond, while ADAMTS3 processes LTBP1 at two distinct sites: P^315^↓A^316^ and P^775^↓A^776^ [33]. ADAMTS6 cleaves both LTBP1 and LTBP3 within their hinge regions [67]. Despite their diverse cleavage specificities, these ADAMTS members collectively converge on a common functional outcome: the disruption of the large latent TGF-β complex, leading to TGF-β release and activation.

Fibulins are major components of elastic fibers in the ECM, imparting elasticity to tissues. This family comprises seven members (fibulin1-7) [68]. Among them, fibulin1 plays roles in hemostasis and vascular development, although its precise functions remain less defined. Fibulin2 regulates the availability of matrix-bound TGF-β by competing with the large latent TGF-β complex for binding sites on FBNs [69]. Fibulin3, 4, and 5 (the “short fibulins”) are crucial for elastic fiber assembly. Specifically, fibulin4 also regulates collagen cross-linking via LOX, and fibulin5 facilitates the interaction between elastin and microfibrils. Fibulin6 and 8 (also known as hemicentin-1 and 2) contribute to basement membrane integrity. Regarding their regulation by ADAMTS proteases, ADAMTS14 mediates the degradation of fibulin2, thereby promoting TGF-β latency [70]. Interestingly, the proteolytic activity of ADAMTS5 toward fibulin2 is antagonized by ADAMTS12, which interacts with fibulin2 without cleaving it [71]. Fibulins can also function as cofactors rather than substrates for ADAMTS proteases. For example, fibulin1 acts as a cofactor for ADAMTS1 by altering the conformation of aggrecan to facilitate its cleavage [72]. Furthermore, recent studies reveal that ADAMTS12 cleaves hemicentin-1 (HMCN1) in the perivascular ECM, enabling the activation and migration of injury-responsive fibroblasts [9]. Overall, the catalytic regulation of fibulins by ADAMTS proteases remains a relatively understudied area.

Collectively, the proteolytic modifications of ECM components by ADAMTS proteases constitute a pivotal upstream regulatory layer in fibrotic remodeling. The excessive deposition of ECM proteins, a hallmark of fibrosis, is not merely a passive outcome but an active driver that perpetuates disease progression through biochemical and mechano-transductive feedback. FN accumulation promotes collagen fibrillogenesis and enhances integrin-mediated cell adhesion [37]. Increased collagen deposition elevates tissue stiffness, which in turn augments mechano-signaling pathways such as FAK and YAP/TAZ [73]. Furthermore, key ECM components including FN, FBN, and LTBP are integral constituents of the latent complex of TGF-β [74]. Their cleavage by specific ADAMTS members releases active TGF-β, thereby initiating and amplifying the core fibrogenic signaling cascade. Thus, ADAMTS proteases orchestrate fibrosis through directly governing the abundance of structural ECM proteins such as collagens, FN, and proteoglycans, and indirectly modulating TGF-β signaling and fibroblast activation, that collectively dictate pathological matrix accumulation. It is important to note, however, that despite considerable substrate overlap in vitro, individual ADAMTS members often exhibit distinct and sometimes opposing roles in fibrosis in vivo. This functional divergence stems from differential substrate preferences among isoforms, spatiotemporal variations in their expression within fibrotic niches, and the pathophysiological context that dictates which ECM substrate dominates at a given stage. Moreover, some proteolytic activities identified in reductionist systems may not fully translate to the complex fibrotic microenvironment, where substrate accessibility, local inhibitors, and cell-matrix interactions further modulate protease function. Therefore, the net effect of an ADAMTS protease in fibrosis depends critically on its specific isoform, tissue context, disease phase, and the dynamic ECM landscape.

## Roles of ADAMTS in fibrotic diseases

ADAMTS proteases exert multifaceted regulatory roles in fibrotic diseases by cleaving key fibrosis-related substrates within the ECM, thereby modulating the TGF-β signaling pathway, collagen deposition and fibroblast plasticity. Recent studies have also revealed that murine models with ADAMTS gene knockouts or mutations exhibit diverse fibrotic phenotypes, highlighting the complex functions of distinct ADAMTS members in organ-specific fibrotic pathologies (Table 1).

**Table 1:** Roles of ADAMTS proteases in fibrosis

Protease	Substrate	Action on substrate	Mechenism in fibrosis	Roles in fibrotic diseases	Phenotype of gene knockout or mutant mice or rats
ADAMTS1	Versican [75, 76]	Degradation[75, 76]	Promote migration of vascular smooth muscle cell in atherogenesis [75]	Atherogenesis(+) [75]	Particularly a significant increase in intimal hyperplasia in ADAMTS1 transgenic/apoE-deficient mice compared with apoE deficiency alone[75]
	LAP-TGF-β [77]	Binds the LKSL motif of LAP promoting its conformational change [77]	Promote release of active TGF-β from LAP-TGF-β in liver fibrosis [77]	Liver fibrosis(+) [77]	
ADAMTS2	Fibrillar procollagen type I-III [23], fibrillar procollagen type Ⅴ [78]	Cleaves the N-pro-peptide of fibrillar collagen [23, 78]	Promote collagen production in liver fibrosis [10]	Liver fibrosis(+) [10]	Reduced extent and stability of CCl_4_-induced hepatic fibrosis and thinner and irregular collagen fibers [10], fragile skin at 1–2 months postnatal, collagen fibrils in skin as bizarre curls in cross-section and decreased diameters of the fibrils [79]
ADAMTS4	Fibronectin [40]	Cleaves in the extradomain A of fibronectin [40]	Mobilize fibronectin-anchored latent TGF-β in heart fibrosis [40]	Cardiac fibrosis(+) [40]	Reduced thickness of left ventricular wall and collagen content in pressure-overloaded rat hearts [40]
ADAMTS5	Aggrecan [80]	Degradation [80]	Disrupt aggrecan-CD44 complex, altering downstream signaling of TGF-β from phosphorylation of smad1/5/8 to smad2/3 in dermal repair [81]	-	Impaired dermal repair in excisional skin wound healing; aggrecan accumulation, altered TGF-β signaling [81]
ADAMTS6	LTBP1, LTBP3 [67]	Cleaves in the hinge region [67]	Cleave LTBP to promote release of active TGF-β in vitro [67]	-	-
	FBN [26]	Degradation [26]	Increase the mechanotension of chondrocytes resulting in an increased translocation of YAP/TAZ to the nucleus in vitro [67]	-	
ADAMTS7	COMP [82, 83]	Degradation [82, 83]	Promote vascular smooth muscle cell migration [82]	Atherogenesis(+) [82]	Substantially ameliorated neointima formation after injury of carotid artery [84], Atherosclerotic aortas of Apoe mice lacking ADAMTS7 contained higher collagen content [85]
	TSP-1 [84, 86]	Degradation [84, 86]	Digest TSP-1 to inhibit re-endothelialization [84]	Atherogenesis(+) [84]	
	TIMP-1 [85]	Degradation [85]	Degrade TIMP-1 reducing its inhibitory effect on MMP-9 which is known to promote collagen degradation to decrease plaque stability [85]	Atherogenesis(+) [85]	
ADAMTS8	OPN [87]	Degradation [87]	Inhibit fibroblast proliferation, migration, and myofibroblast trans-differentiation and induce apoptosis [8]	Hypertrophic scar(-) [8]	-
ADAMTS12	HMCN1 [9]	Degradation	Enable the activation and migration of a distinct injury-responsive fibroblast subset defined by aberrant high JAK/STAT signaling [9]	Heart fibrosis(+), renal fibrosis(+) [9]	Ameliorated fibrosis in kidney and heart respectively after UUO surgery and MI surgery [9]
ADAMTS13	vWF [25]	Degradation	Curb vWF-mediated platelet recruitment and inhibit subsequent release of active TGF-β [88]	Cardiac fibrosis(-) [88]	-
ADAMTS14	Fibrillar procollagen type I-III [23], fibrillar procollagen type Ⅴ [78]	Cleaves the N-pro-peptide of fibrillar collagen [23, 78]	Process procollagen, contributing to excessive collagen production [5]	Dupuytren’s disease(+) [5]	-
ADAMTS16	LAP [6, 89]	Binds LAP promoting its conformational change [6, 89]	Promote the release of active TGF-β in cardiac and renal fibrosis [6, 89]	Cardiac fibrosis(+) [6], renal fibrosis(+) [89]	Accentuated fibrosis and dysfunction of the pressure-overloaded heart [6]
ADAMTS18	LAP [90]	Binds the LSKL motif of LAP promoting its conformational change [90]	Promote the release of active TGF-β in renal fibrosis [90]	Renal fibrosis(+) [90]	Unilateral or bilateral SMG scleroma that is similar to patients with chronic sclerosing sialadenitis of the submandibular gland [91], preputial gland hypoplasia and fibrosis in male mice [92], pathological changes of lacrimal gland including acinar atrophy and irregular duct ectasia with periductal fibrosis [93]
	Fibronectin [27]	Cleaves in the N-terminal linker sequence [27]	Degrade fibronectin decreasing adhesion formation to inhibit FAK activation and AKT/Notchsignaling [7, 94]	Renal fibrosis(-) [94]	

*LAP* Latency-associated peptide, *LTBP* Latent TGF-β binding protein, *TGF-β* Transforming growth factor-β, *OPN* Osteopontin, *vWF* von Willebrand factor, *COMP* Cartilage oligomeric matrix protein, *TSP-1* Thrombospondin-1, *HMCN1* Hemicentin1, *UUO* Unilateral ureter obstruction, *MI* Myocardial infarction, *SMG* Submandibular salivary gland

### Regulation of TGF-β signaling

**Fig. 6:**
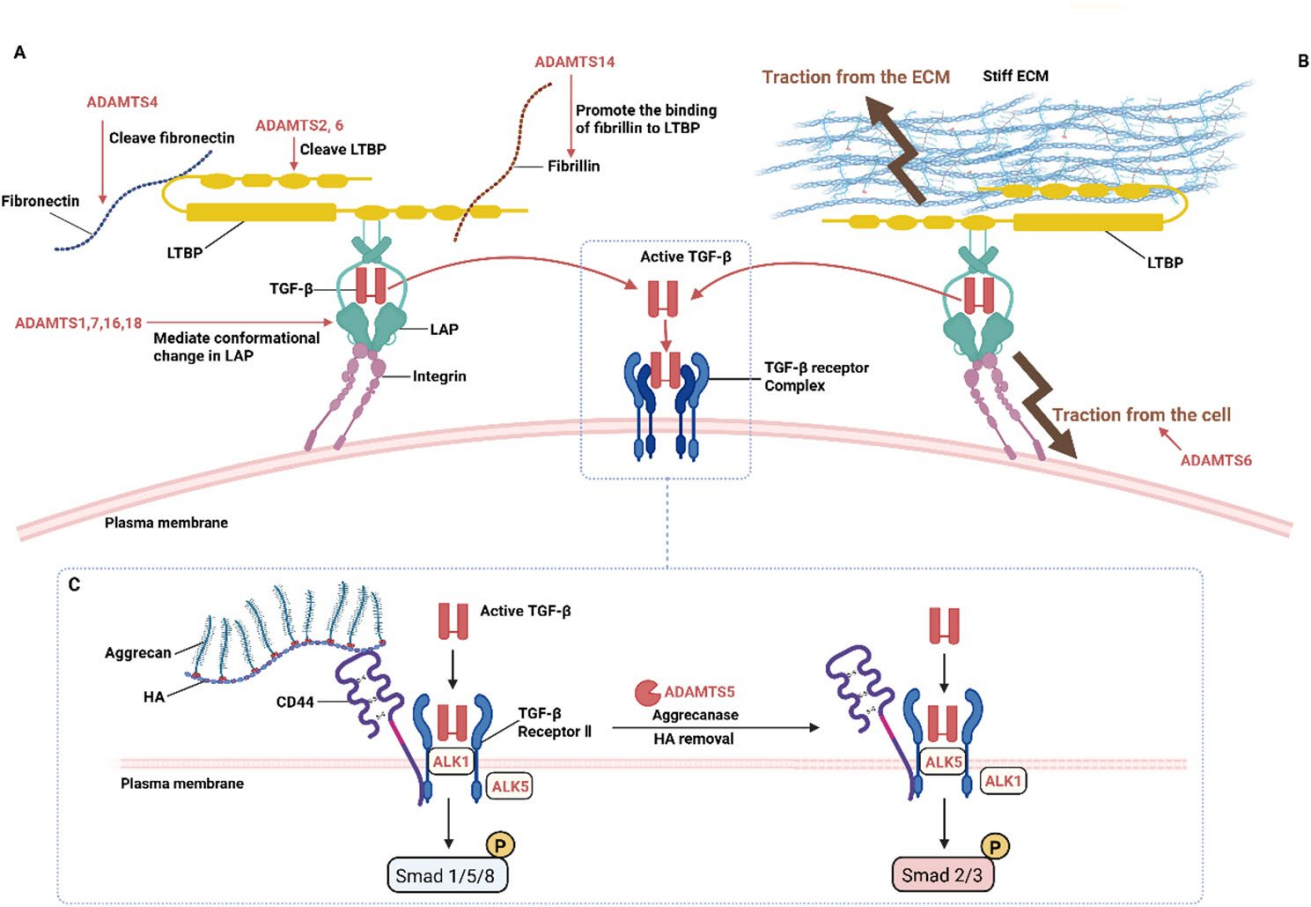
Multifaceted regulation of TGF-β signaling by ADAMTS proteases through ECM remodeling. **A** ADAMTS proteases exert enzymatic regulation over the latent TGF-β complex. ADAMTS1/7/16/18 bind directly to LAP, inducing conformational changes that release active TGF-β; ADAMTS2/6 cleave LTBP, disrupting ECM anchorage of the large latent complex; ADAMTS4 cleaves fibronectin in its EDA domain, mobilizing matrix-bound latent TGF-β. ADAMTS14 indirectly inhibits TGF-β bioavailability by competitively displacing fibulin2 from fibrillins, thereby stabilizing ECM anchorage of the latent TGF-β complex. **B** ADAMTS proteases contribute to the mechanical activation of TGF-β by modulating ECM stiffness and cellular contractility. For instance, ADAMTS6 elevates cellular tension, amplifying the mechanical force transmitted across the integrin-bound LAP and facilitating force-dependent conformational release of active TGF-β. **C** Modulation of TGF-β signaling through aggrecan cleavage. ADAMTS5-mediated aggrecanolysis disrupts aggrecan-HA-CD44 complexes. This alters TGF-β receptor signaling balance, promoting Smad1/5/8 phosphorylation via ALK1, while suppressing Smad2/3 phosphorylation via ALK5. ECM: Extracellular matrix; LAP: Latency-associated peptide; LTBP: Latent TGF-β binding protein; HA: Hyaluronic acid; TGF-β: Transforming growth factor-β. This schematic was adapted from Refs [73, 81]. The figure was prepared using BioRender (https://www.biorender.com/)

TGF-β is a major driver of fibroblast transdifferentiation and fibrotic diseases pathogenesis [74]. Initially, TGF-β exists as an inactive dimer sequestered in the ECM [74]. It binds to latent TGF-β binding protein (LTBP) via the latency-associated peptide (LAP), forming a large latent complex (LLC) [74]. LAP encapsulates TGF-β dimer to form the LAP-TGF-β complex, preventing TGF-β from binding to its receptor [95]. The binding of LAP with LTBP is mediated by a pair of disulfide bonds between the most amino-terminal cysteines in LAP and a unique cysteine pair in LTBP [96]. This LLC further associates with ECM components such as fibronectin fibers and fibrillin microfibrils, resulting in an even larger complex [95]. Specifically, LTBP1 binds to FN primarily through an interaction site within type III domains 12-14 of FN, with additional stabilization provided by the EDA domain [97, 98]. In addition, the last three carboxy-terminal domains of LTBP1 interact with the amino terminus of FBN [96]. The key of TGF-β activation is the release of active TGF-β from the latent complex composed of LAP-TGF-β, LTBP, FN and FBN in the ECM. It is regulated either by direct proteolysis or conformational change of the components of the complex, or by increasing the mechano-tension of the cell attached to the ECM. ADAMTS proteases play a pivotal role in both regulatory mechanisms (Fig. 6).

ADAMTS proteases exert enzymatic regulation over the latent TGF-β complex. The main components of the complex including LAP, LTBP, FN and FBN, are all substrates of ADAMTS proteases. ADAMTS1, 7, 16 and 18 can directly interact with LAP to promote the release of bioactive TGF-β, primarily through inducing conformational changes. Bourd-Boittin et al. identified ADAMTS1 as a critical mediator in liver fibrogenesis by directly engaging with LAP [77]. In the hepatic stellate cell, ADAMTS1 is synthesized as a 110 kDa latent form and processed into an 87 kDa mature form that accumulates in fibrotic tissues [77]. ADAMTS1 directly interacts with the LKSL motif of LAP-TGF-β through its KTFR motif near its non-carboxy-terminal TSP1, promoting a conformational change in LAP to induce TGF-β release, and the WGPW peptide near the ADAMTS1 TSP1 motif facilitates this effect [77]. Similarly, ADAMTS16 interacts with LAP-TGF-β through its RRFR motif, promoting the release of active TGF-β, which is a key regulatory event driving progression in cardiac and renal fibrosis [89, 99]. These findings suggest that therapeutic monoclonal antibodies targeting the RRFR motif of ADAMTS16 may represent a promising strategy to inhibit fibrosis [99]. ADAMTS18 has been identified as a novel and critical protease that exacerbates renal fibrosis by directly binding to and activating latent TGF-β. Its conserved KPFR motif within the TSR domain (residues 589–669) competitively binds to the LSKL sequence at the N-terminus of LAP, disrupting the LAP-mature TGF-β interaction and thereby directly promoting the release of active TGF-β1 and activating the downstream TGF-β1/Smad signaling pathway to drive fibrosis. ADAMTS18 expression is specifically upregulated in fibrotic kidneys of patients with chronic kidney disease, which highlights the unique pathogenic role of ADAMTS18 in renal fibrosis [90]. ADAMTS7 and ADAMTS16 are upregulated to similar levels in the murine model induced by transverse aortic constriction [99]. These two proteases share homologous amino acid sequences at their WxxW and KRFK/RRFR motifs, suggesting that ADAMTS7 may similarly modulate LAP conformation to facilitate TGF-β activation [100].

In addition to direct interaction with LAP, ADAMTS proteases also regulate TGF-β bioavailability by targeting the structural components that anchor the latent complex including LTBP, FN, and FBN. Cleavage of these anchors promotes the release of the LLC from the ECM, thereby facilitating subsequent processing by other proteases (e.g., MMPs, plasmin) that cleave LAP to liberate active TGF-β. ADAMTS2 in human dermal fibroblasts downregulates the cellular response to both TGF-β1 and TGF-β2 due to its cleavage to LTBP1 [33]. This regulatory mechanism has been validated in the context of liver fibrosis, where ADAMTS2 promotes TGF-β signaling in vivo and is recognized as a risk factor for hepatic fibrogenesis [10, 101]. ADAMTS6 can also cleave LTBP3 as well as LTBP1, and bind to their respective large latent TGF-β complexes to release effective TGF-β in vitro [67]. ADAMTS4 mobilizes ECM-anchored latent TGF-β by cleaving the EDA domain of FN, which promotes cardiac fibrosis [40]. It is demonstrated that ADAMTS4 treatment increases the level of ECM-unanchored TGF-β by approximately 50% in the culture medium of human cardiac fibroblasts. Furthermore, in failing human hearts, a marked increase is observed in ADAMTS4 expression and its cleavage activity. ADAMTS14 downregulates TGF-β bioavailability due to its interaction with fibulin2 [102]. Indirect regulation via competitive displacement is exemplified by ADAMTS14. ADAMTS14 interacts with fibulin2, and its knockdown paradoxically enhances TGF-β responses. This effect is reversed by co-knockdown of fibulin2, leading to the model that in the absence of ADAMTS14, elevated fibulin2 outcompetes the LLC for binding sites on FBN, thereby disrupting ECM anchorage and increasing TGF-β bioavailability [102].

Beyond regulating compartmentalization of latent TGF-β, ADAMTS proteases are also involved in the mechano-activation of TGF-β. This occurs through two interconnected mechanisms: altering ECM composition and stiffness, and regulating cellular contractility. Together, these changes enhance the mechanical tension transmitted across integrin-bound latent complexes. This force can induce a conformational change in the LAP, leading to the liberation of active TGF-β [96]. On one hand, ADAMTS proteases impact ECM stiffness by modulating matrix composition including collagen deposition. On the other hand, they modulate cellular contractility by regulating fibroblast activation. A representative example is provided by ADAMTS6. Its overexpression in chondrocytes significantly elevates cellular tension, as evidenced by activation of the Hippo-YAP/TAZ pathway [67]. This amplifies the mechanical force transmitted across the integrin-bound LAP, facilitating force-dependent conformational release of active TGF-β. The detailed roles of ADAMTS proteases in regulating ECM stiffness and fibroblast contractility will be elaborated in the following sections.

Apart from their primary role in regulating the bioavailability of active TGF-β, ADAMTS proteases can modulate TGF-β signaling through additional pathways. ADAMTS5 regulates downstream TGF-β signaling without affecting the level of active TGF-β. Dermal fibroblasts from ADAMTS5 knockout mice showed suppressed pSmad2/3 signaling but upregulated pSmad1/5/8 signaling upon TGF-β1 stimulation, which was attributed to aggrecan accumulation in the ECM [81]. This altered signaling pattern was reversed either by CD44 knockout or by treatment with *Streptomyces* hyaluronidase, which degrades aggrecan [81]. Since aggrecan is known to interact with the cell surface receptor CD44 to modulate intracellular signaling, these findings suggest that ADAMTS5-mediated cleavage of aggrecan disrupts the aggrecan-CD44 interaction [103]. This disruption, in turn, selectively promotes Smad2/3 phosphorylation while suppressing Smad1/5/8 phosphorylation in response to TGF-β1. ADAMTS proteases can also modulate TGF-β signaling in a distinct manner. ADAMTS13 is primarily recognized for its specific proteolytic activity toward vWF. Witsch et al. demonstrated that recombinant human ADAMTS13 inhibits cardiac vWF deposition and attenuates platelet recruitment into the myocardium following left ventricular pressure overload in mice. This intervention concomitantly reduced plasma levels of TGF-β1, despite leaving total TGF-β1 largely unchanged [88]. Given that platelets serve as a major reservoir of TGF-β1, it is suggested that ADAMTS13, by cleaving vWF, limits vWF-mediated platelet recruitment and subsequent release of active TGF-β [88]. ADAMTS12 appears to enhance TGF-β signaling through a mechanism that remains unclear [104]. In hepatic stellate cells, knockdown of ADAMTS12 led to the downregulation of canonical TGF-β-responsive genes (e.g., PAI-1) and induced a less differentiated cellular phenotype, characterized by an altered actin network and decreased nuclear spreading. Importantly, these molecular and phenotypic changes were all rescued by exogenous TGF-β treatment, reinforcing the conclusion that ADAMTS12 functions as a positive regulator of the TGF-β signaling pathway.

Acting as more than mere processors of the latent TGF-β complex, members of the ADAMTS family are intricately integrated into the feedback regulatory circuits that fine-tune and often amplify TGF-β signaling, thereby critically shaping the progression of fibrosis. These proteases often function within positive feedback loops, acting both as critical downstream effectors that execute TGF-β activation and as direct transcriptional targets whose expression is upregulated by TGF-β signaling itself. This dual role creates a self-reinforcing circuit that sustains and escalates pathway activity. A prime example is ADAMTS2. TGF-β1 potently induces ADAMTS2 gene expression at the transcriptional level [105]. In turn, ADAMTS2 processes components of the large latent TGF-β complex, facilitating the release of bioactive TGF-β [33]. This establishes a self-reinforcing cycle: initial TGF-β activation upregulates ADAMTS2, which then processes the latent complex to generate more active TGF-β, thereby amplifying the fibrotic signal. This ADAMTS2-mediated positive feedback loop has been implicated in promoting TGF-β signaling and fibrogenesis in contexts such as liver fibrosis. Similarly, ADAMTS16 also forms a positive feedback axis [106]. It interacts with LAP-TGF-β1 to promote its activation, and the resulting TGF-β/Smad signaling upregulates the transcription factor SOX4, which directly binds to and transactivates the ADAMTS16 promoter, creating a potent ADAMTS16/TGF-β1/SOX4 circuit that drives tumor progression. Furthermore, ADAMTS12 modulates hepatic stellate cell activation downstream of TGF-β, and its loss can trigger a compensatory upregulation of TGF-β pathway activity, suggesting its role within a complex regulatory network that may involve adaptive feedback mechanisms [9, 104]. These examples underscore that ADAMTS proteases are active, dynamic amplifiers within TGF-β signaling networks. By forming context-dependent positive feedback loops, they convert transient TGF-β stimuli into sustained signaling outputs, a feature central to the pathogenesis of fibrosis.

### Regulation of collagen deposition

**Fig. 7:**
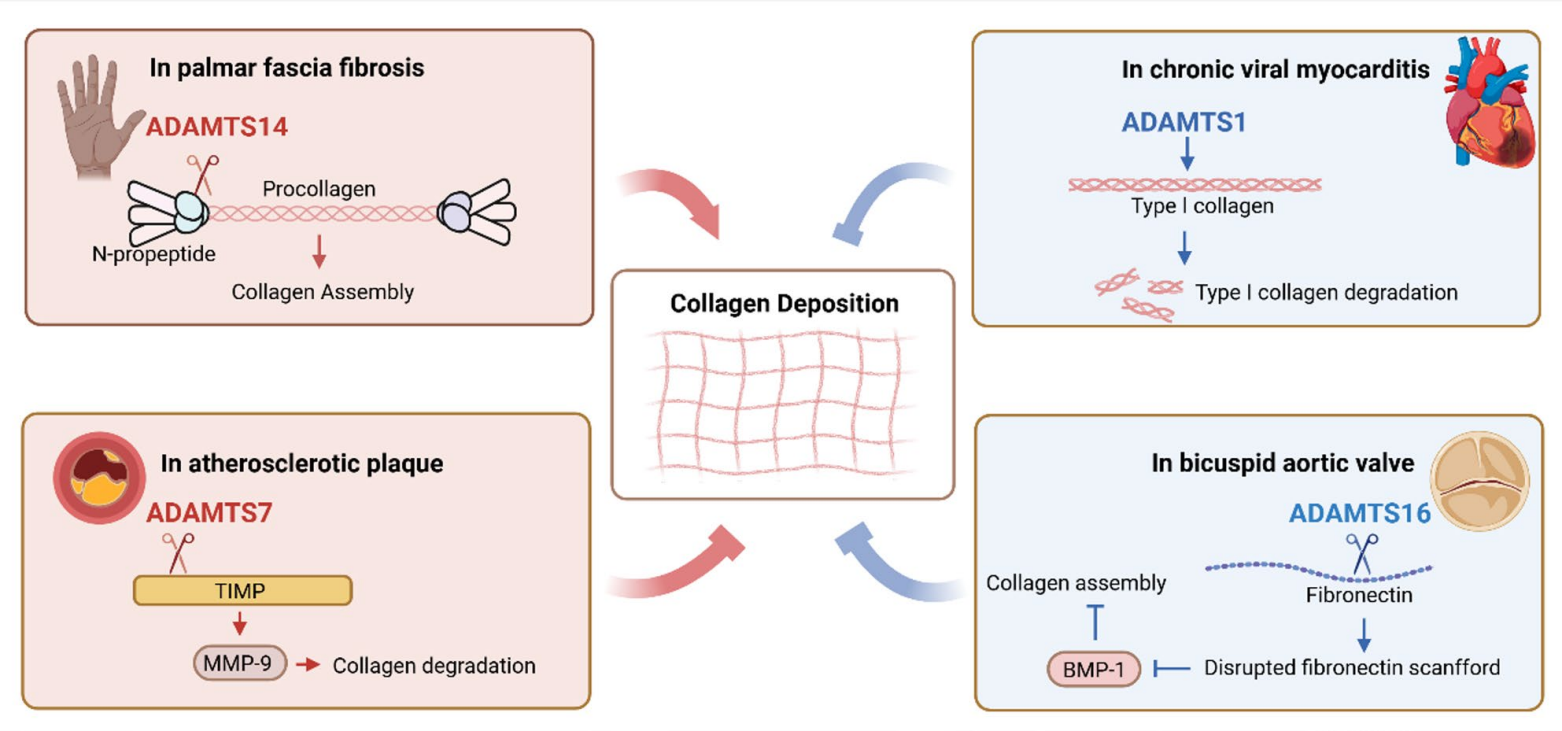
Roles of ADAMTS proteases in regulation of collagen deposition across fibrosis related diseases. In palmar fascia fibrosis, upregulated ADAMTS14 processes procollagen to mature collagen, promoting excessive fibrillar collagen deposition. In atherosclerotic plaque formation, ADAMTS7 degrades TIMP-1, alleviating inhibition of MMP-9 and thereby enhancing collagen degradation and decreasing plaque stability. In chronic viral myocarditis, ADAMTS1 contributes to the degradation of type I collagen. In bicuspid aortic valve, loss of ADAMTS16 disrupts fibronectin matrix organization, impairing BMP-1 mediated procollagen processing and leading to aberrant collagen assembly and ECM disorganization. These highlight how the net effect of ADAMTS activity on collagen accumulation is determined by the specific protease, disease context, and substrate availability. ECM: Extracellular matrix; TIMP-1: Tissue inhibitor of metalloproteinases-1; MMP-9: Matrix metalloproteinase-9; BMP-1: Bone morphogenetic protein 1. The figure was prepared using BioRender (https://www.biorender.com/)

Excessive accumulation of fibrillar collagens is one of the most distinctive characteristics of fibrosis [107]. ADAMTS proteases also act as direct regulators of collagen deposition in multiple fibrotic diseases (Fig. 7). Increased collagen biosynthesis mediated by elevated procollagen N-peptidase ADAMTS14 is a key factor in the pathogenesis of Dupuytren’s disease, a common fibrotic disorder of the palmar fascia [5]. This suggests that inhibiting ADAMTS14 could attenuate excessive collagen production [5]. The functional redundancy among the three previously described procollagen N-peptidases (ADAMTS2, ADAMTS3, and ADAMTS14) raises the possibility of selectively inhibiting ADAMTS14 without completely disrupting global collagen biosynthesis [5]. Therefore, targeting ADAMTS14 may be sufficient to slow or prevent collagen deposition in Dupuytren’s disease.

ADAMTS16 and ADAMTS18 inhibit collagen assembly through their proteolytic activity to FN. In cardiac valves, loss of ADAMTS16 leads to not only aberrant accumulation of its substrate FN but also excessive collagen deposition, resulting in disruption of ECM [108]. It has been established that FN serves as an essential scaffold for the binding of procollagen C-peptidase (BMP-1) and subsequent collagen processing [109]. Thus, it is suggested that ADAMTS16 regulates collagen fibril assembly through modulation of FN organization. It is indicated that ADAMTS18 has the same effect [7]. In vitro, ADAMTS18 cleaves FN, and in vivo ADAMTS18 deletion causes increased collagen deposition.

ADAMTS1 and ADAMTS7 regulate collagen deposition in the ECM. Guo et al. revealed an association between ADAMTS1 and type I collagen degradation in the murine model of chronic viral myocarditis, a condition characterized by pathological accumulation of type I collagen in cardiac tissue [110]. ADAMTS1 expression levels exhibited an inverse correlation with type I collagen levels and a positive correlation with levels of the C-terminal telopeptide of type I collagen, which is a marker of collagen degradation [110]. Notably, by accelerating type I collagen degradation, ADAMTS1 contributes to the anti-fibrotic effects of captopril, an angiotensin converting enzyme inhibitor (ACEi) frequently used in clinical practice [110]. Although the precise underlying mechanism remains to be fully elucidated, the interplay between ACEi and ADAMTS1 suggests promising strategies for combination anti-fibrotic therapies [110]. ADAMTS7 also inhibits collagen deposition in the ECM. Specifically, co-immunoprecipitation assays demonstrate that the catalytic domain of ADAMTS7 binds to tissue inhibitor of metalloproteinases-1 (TIMP-1) and mediates its degradation [85]. By reducing TIMP-1 stability, ADAMTS7 attenuates TIMP-1 mediated inhibition of its canonical target, matrix metalloproteinase-9 (MMP-9), which has been known to facilitate collagen degradation [85]. This weakens the stability of plaques in atherosclerosis.

### Regulation of fibroblast plasticity

**Fig. 8:**
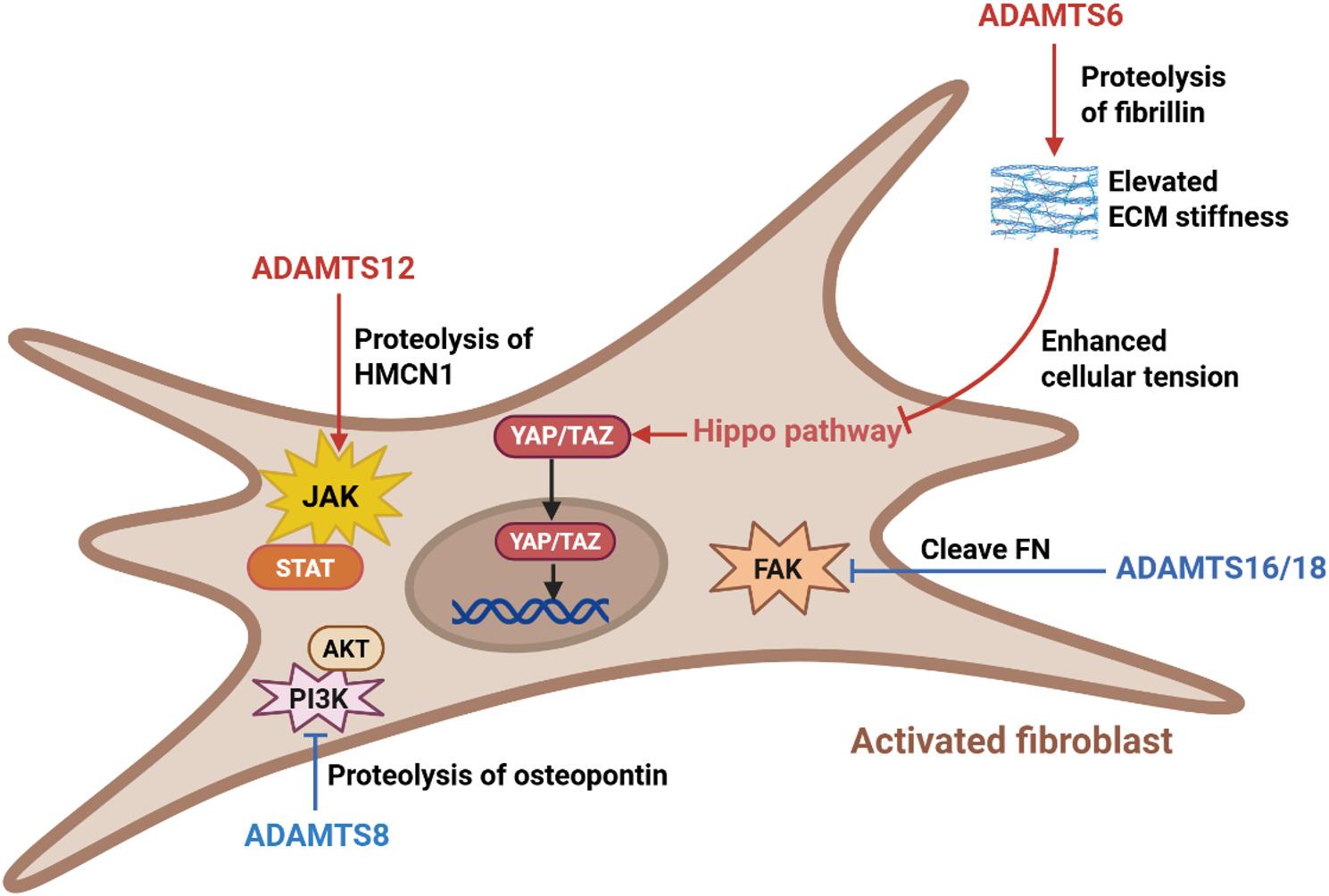
ADAMTS proteases modulate key pathways driving fibroblast plasticity and fibrogenesis. ADAMTS12 cleaves HMCN1, promoting fibroblast migration and activating pro-fibrotic JAK-STAT signaling. ADAMTS6 promotes fibroblast activation by degrading fibrillin and altering the mechanical properties of the ECM, which inactivates the Hippo pathway and enables nuclear translocation of YAP/TAZ to drive fibroblast activation. ADAMTS16 and ADAMTS18 regulate fibroblast adhesion and phosphorylation of FAK via proteolytic cleavage of fibronectin. ADAMTS8 inhibits PI3K-AKT pathway through its proteolytic activity on OPN. ECM: Extracellular matrix; FAK: Focal adhesion kinase; HMCN1: Hemicentin-1; JAK-STAT: Janus kinase-signal transducer and activator of transcription pathway; FN: Fibronectin; OPN: Osteopontin. The figure was prepared using BioRender (https://www.biorender.com/)

Fibroblasts exhibit remarkable plasticity during fibrotic progression and resolution [111]. Following injury, fibroblast progenitors migrate to the lesion site, where they contribute to tissue repair primarily through ECM deposition. If this process becomes dysregulated, it drives fibrotic progression, concomitant with the sustained activation of intracellular signaling pathways. The migration of these precursor cells to the wound area, along with their accompanying phenotypic transformation, requires the precise hydrolysis of specific ECM components. Moreover, fibroblasts possess the ability to sense both the mechanical properties and biochemical composition of the ECM, allowing them to dynamically regulate their own phenotype [73]. For instance, fibroblasts engage with ECM components such as FN and collagens via integrins. Under mechanical stress, such as that imposed by a stiffened ECM, these interactions mediate firm cell adhesion. This adhesion not only provides mechanical anchorage but also transduces biochemical signals that promote fibroblast proliferation, activation, and migration. Indeed, increased ECM stiffness has been shown to significantly enhance fibroblast activation. Furthermore, specific biochemical cues within the ECM, such as the glycoprotein osteopontin (OPN), can also critically modulate fibroblast phenotype and behavior [112]. ADAMTS proteases play crucial roles in orchestrating these interconnected processes (Fig. 8).

Several ADAMTS proteases, including ADAMTS1, ADAMTS7, and ADAMTS12, promote fibroblast migration through the proteolysis of specific ECM substrates. A prime example involves Gli1⁺ perivascular cells, a minor population that serves as a major source of organ myofibroblasts [113, 114]. Upon injury, these cells expand, migrate from perivascular niches into the interstitium, and differentiate into myofibroblasts, a process often accompanied by microvascular damage [115]. Using an unbiased profiling approach, Hoeft et al. identified ADAMTS12 as a fibroblast-specific gene that is strongly upregulated during fibrotic remodeling in human and murine kidneys and hearts [9]. In vivo studies confirmed that ADAMTS12 is critical for fibroblast activation and fibrogenesis, as its genetic deletion in mice attenuates renal and cardiac fibrosis and preserves cardiac function post-injury. Mechanistically, the authors combined spatial transcriptomics, CRISPR/Cas9 gene editing, and catalytic variant analyses to demonstrate that ADAMTS12 cleaves HMCN1, thereby driving fibroblasts into an injury-responsive state characterized by enhanced migration. This state exhibits aberrantly elevated JAK/STAT signaling, analogous to the pro-fibrotic signaling observed in myeloproliferative neoplasms [116]. Mirroring the role of ADAMTS12, ADAMTS1 promotes fibroblast migration and phenotypic switching in contexts such as kidney injury, skin repair, and atherosclerosis. For instance, ADAMTS1 drives pericyte detachment from peritubular capillaries and promotes their differentiation into scar-forming myofibroblasts in the kidney [117]. During cutaneous wound healing, it facilitates fibroblast migration to the wound site through its proteolytic activity [118]. Similarly, ADAMTS1 promotes the migration of vascular smooth muscle cells (VSMCs), which can undergo fibroblastic conversion in atherosclerosis [75]. Although the precise ECM substrate remains unclear, versican has been proposed as a candidate target of ADAMTS1 in these processes. Likewise, ADAMTS7 mediates VSMC migration through the degradation of COMP within the ECM [82].

ADAMTS16 and ADAMTS18 regulate fibroblast adhesion and focal adhesion kinase (FAK) phosphorylation through the proteolytic cleavage of FN. As a key ECM glycoprotein containing multiple integrin-binding sites, FN degradation by these proteases directly inhibits fibroblast adhesion. Moreover, FN cleavage impairs collagen assembly, as previously mentioned, leading to reduced ECM stiffness. In vivo evidence supports this mechanism: aortic valves from ADAMTS16-knockdown mice exhibited thickening, accompanied by FN accumulation, elevated FAK phosphorylation, and excessive fibroblast proliferation, with significant colocalization observed between FN and phosphorylated FAK [108]. Similarly, ADAMTS18 cleaves FN in vitro, and its genetic deletion in vivo results in increased collagen deposition, impaired Hippo signaling, and enhanced nuclear translocation of YAP/TAZ [7]. These findings provide a compelling mechanistic basis for the protective role of ADAMTS18 in fibrosis [94]. Consistently, Xu et al. demonstrated that ADAMTS18 knockdown exacerbates renal interstitial ECM accumulation, and that TGF-β-mediated methylation downregulates ADAMTS18 expression, thereby promoting renal fibrosis through potentiation of the AKT/Notch pathway. This may also be mechanistically linked to FAK function, as phosphorylation at key tyrosine residues can create a major docking site for PI3K regulatory subunits, directly activating the PI3K/AKT signaling axis [119].

ADAMTS6 promotes fibroblast activation by degrading FBN and altering the mechanical properties of the ECM. Specifically, FBN degradation disrupts elastic fiber assembly and increases ECM stiffness [67]. Mechanistically, while fibrillar collagens primarily confer tissue strength, an increase in the microfibril content tends to render the matrix more compliant. By degrading FBN and reducing microfibrils, ADAMTS6 contributes to a stiffer mechanical microenvironment. This elevated stiffness enhances cellular mechanical tension, which inactivates the Hippo signaling pathway. Consequently, the YAP/TAZ complex translocates to the nucleus and drives the transcriptional program of fibroblast activation.

ADAMTS8 acts as a negative regulator of fibroblast proliferation, migration, and myofibroblast differentiation, primarily through its proteolytic activity against OPN [8, 87]. Supporting this role, Ma et al. identified a highly expressed long non-coding RNA in hypertrophic scar tissue, which they designated hypertrophic scar fibroblast-associated lncRNA (HSFAS). Overexpression of HSFAS promoted fibroblast proliferation, migration, myofibroblast transdifferentiation, and suppressed apoptosis. Importantly, HSFAS was found to repress ADAMTS8 expression, and knockdown of ADAMTS8 rescued the cellular phenotypes suppressed by HSFAS downregulation, functionally linking HSFAS to ADAMTS8. Mechanistically, ADAMTS8 cleaves and promotes the degradation of OPN, a glycoprotein known to signal through αvβ3 integrin on fibroblasts to promote activation, collagen deposition, and fibrosis via the PI3K-Akt pathway [112]. Thus, by degrading OPN, ADAMTS8 inhibits its pro-fibrotic signaling.

## Shared mechanisms and functional divergence of the ADAMTS proteases in fibrosis

The functional impact of ADAMTS proteases in fibrosis predominantly arises from their proteolytic processing of key ECM components. This activity directly regulates the abundance and architecture of structural ECM proteins and, importantly, modulates the biochemical and mechanical signaling properties of the matrix. By altering the ECM composition, ADAMTS members indirectly influence fibroblast behavior, thereby creating a dynamic feedback loop that further governs ECM deposition. Notably, several functional themes are shared across distinct family members through the targeting of common substrates. For instance, ADAMTS1, 7, 16, and 18 converge on the latent TGF-β complex by directly binding to the LAP, a shared mechanism that promotes TGF-β activation and drives fibrogenesis [6, 90, 120]. Similarly, ADAMTS2 and 6 cleave LTBP, while ADAMTS2 and 14 process procollagen N-propeptide, each action contributing to enhanced TGF-β signaling or collagen assembly, respectively [10, 33, 67, 121]. In contrast, ADAMTS16 and 18 share the substrate FN; their cleavage disrupts FN matrix organization, which in turn inhibits collagen fibrillogenesis and attenuates fibroblast adhesion and FAK signaling, collectively exerting an anti-fibrotic effect.

The pathophysiological role of an individual ADAMTS protease is often the net result of its multi-substrate activity, where proteolytic events may have synergistic or opposing effects on the fibrotic cascade. A prime example is ADAMTS2, which cleaves both LTBP and procollagen. The former promotes TGF-β bioavailability, and the latter facilitates mature collagen deposition, creating a coordinated pro-fibrotic program. Conversely, ADAMTS7 exhibits a more complex, dualistic function: it can promote fibrosis by interacting with LAP-TGF-β, yet simultaneously degrades TIMP-1, which alleviates inhibition of MMP-9 and subsequently enhances collagen degradation [6, 85]. This functional pleiotropy underscores that the overall contribution of an ADAMTS member to fibrosis is not defined by a single substrate but by the integrated outcome of its proteolytic repertoire. Currently, most identified ADAMTS members predominantly exert pro-fibrotic actions. Only a subset, including ADAMTS7, 8, 16 and 18, has been clearly demonstrated to possess context-dependent protective or anti-fibrotic functions, highlighting the family’s overall inclination toward driving pathological matrix accumulation [7, 8, 27, 28].

Functional divergence among ADAMTS proteases is fundamentally rooted in their exquisite substrate specificity. Certain members exert unique roles by hydrolyzing highly selective substrates that are not broadly targeted by the family. For example, ADAMTS12 specifically cleaves HMCN1 to enable fibroblast migration, ADAMTS7 uniquely degrades TIMP-1, ADAMTS8 preferentially processes OPN to inhibit fibroblast activation, and ADAMTS14 interacts with and cleaves fibulin2 to modulate TGF-β latency [8, 9, 70, 85]. This precise substrate targeting allows individual ADAMTS proteases to engage distinct nodes within the complex fibrotic network. Divergence also arises from differential substrate affinity and cleavage site preference, even when enzymes share a common substrate. Although ADAMTS2, like ADAMTS16 and 18, possesses the capability to cleave FN, it does not recapitulate their anti-fibrotic phenotype. This is likely because ADAMTS2 exhibits a stronger catalytic preference and affinity for LAP and LTBP, directing its primary activity toward the TGF-β activation axis rather than FN matrix disruption. Similarly, both ADAMTS4 and ADAMTS16/18 can proteolyze FN, but they target different domains with opposing functional consequences. ADAMTS4 cleaves within the EDA domain, destabilizing the FN-anchored latent TGF-β complex and promoting TGF-β release, thereby driving fibrosis [40]. In contrast, ADAMTS16 and 18 cleave within the N-terminal heparin-binding region, which impairs cell-ECM adhesion and microfibril assembly, ultimately inhibiting collagen deposition.

Collectively, the ADAMTS family orchestrates fibrotic remodeling through a sophisticated interplay of shared mechanistic themes and member-specific specializations. Their actions are integrated at the level of ECM composition, growth factor bioavailability, and cellular responsiveness. The final pro- or anti-fibrotic outcome for a given ADAMTS protease is therefore not predetermined but is dynamically shaped by its specific substrate portfolio, relative catalytic efficiencies, and the pathophysiological context that determines which substrates are available and dominant at a given time and location within the evolving fibrotic lesion.

## Targeting ADAMTS as a therapeutic strategy for fibrosis

Existing studies have identified several exogenous and endogenous factors capable of targeting ADAMTS proteases and modulating fibrotic processes (Table 2). A central endogenous regulator of ADAMTS proteases in ECM homeostasis is tissue inhibitor of metalloproteinase (TIMP)-3. Unlike other TIMPs, TIMP-3 is a unique, potent inhibitor of several ADAMTS members, most notably the aggrecanases ADAMTS4 and ADAMTS5, with inhibition constants in the subnanomolar range [122]. The inhibitory interaction is not confined to the catalytic domain. The C-terminal ancillary domains of ADAMTS4 and ADAMTS5 significantly enhance their affinity for the N-terminal domain of TIMP-3, suggesting an allosteric or exosite-mediated regulation [123]. This interaction can be further modulated by the ECM microenvironment. For instance, the presence of the native substrate aggrecan, via its chondroitin sulfate chains, binds to the spacer domain of ADAMTS4 and forms a ternary complex that markedly increases the enzyme’s affinity for TIMP-3 [124]. The physiological significance of TIMP-3 extends beyond cartilage, as it is also expressed in the central nervous system white matter alongside ADAMTS1, 4, and 5, where an imbalance (e.g., decreased TIMP-3 with increased ADAMTS4) is implicated in pathological ECM turnover in conditions like multiple sclerosis [125]. Furthermore, TIMP-3’s regulatory repertoire includes inhibiting other ADAMTS family members such as the procollagen N-proteinase ADAMTS2, highlighting its broad role in collagen biosynthesis and fibrillogenesis [126]. Crucially, the extracellular levels of TIMP-3 are dynamically controlled by endocytic clearance via the receptor low-density lipoprotein receptor-related protein (LRP)-1 [127]. The disease-modifying agent pentosan polysulfate (PPS) exerts a multifaceted inhibitory effect on ADAMTS-driven pathology precisely by targeting this TIMP-3 axis. PPS not only acts as a direct exosite inhibitor of ADAMTS4 and 5 but also potently increases ECM TIMP-3 levels by blocking its LRP-1-mediated endocytosis [128, 129]. Moreover, PPS functions as a molecular bridge, forming an electrostatically driven trimolecular complex with ADAMTS5 and TIMP-3, thereby enhancing their binding affinity by over 100-fold and locking the protease in an inhibited state [128, 129]. In a rat model of aortic banding, treatment with PPS significantly improved cardiac contractile function, as evidenced by increased fractional shortening (vehicle: 48 ± 3% vs. PPS: 60 ± 1%; *p* < 0.01) [130]. Furthermore, PPS administration resulted in an approximately 80% reduction in myocardial ADAMTS4 mRNA expression and a nearly 50% decrease in extracellular versican cleavage fragments, indicating suppressed versicanase activity [130].

**Table 2:** Targeting ADAMTS as a therapeutic strategy for fibrosis

Agent	Target	Model/Disease	Effect	References
Pentosan polysulfate	ADAMTS4	Rat, pressure-overloaded heart	Improved heart systolic function	[130]
Hydroxamate-based small molecule designated ‘13n’	ADAMTS4	Rat, pressure-overloaded heart	Improved systolic function, decreased myocardial collagen content	[40]
Extracellular matrix protein 1	ADAMTS1	Mouse, damaged liver	Attenuated liver fibrosis	[131]
LSKL peptide	ADAMTS18	Rat, ureteral obstruction kidney	Attenuated kidney fibrosis	[90]
Recombinant human ADAMTS13	ADAMTS13	Mouse, pressure-overloaded heart	Improved cardiac function, attenuated heart fibrosis	[88]

Despite the shared proteolytic activities between ADAMTS4 and ADAMTS5, inhibition of ADAMTS5 was found to exacerbate cardiac dysfunction in the murine model of pressure overload [132]. Previous studies have shown that PPS interacts with the noncatalytic spacer domain of ADAMTS4 and the cysteine-rich domain of ADAMTS5, blocking activity of both ADAMTS4 and ADAMTS5 [128]. To achieve more selective inhibition, Vistnes et al. investigated the hydroxamate-based small molecule ‘13n’, which exhibits markedly higher specificity for ADAMTS4 (IC₅₀ = 26 nM) compared to ADAMTS5 (IC₅₀ = 860 nM) [40, 133]. Rats receiving 13n showed substantially better cardiac function than vehicle-treated rats, including ∼30% reduction in E/e’ and left atrial diameter, indicating an improvement in diastolic function [40]. 13n treatment also resulted in a marked reduction in myocardial collagen content and a down-regulation of TGF-β target genes, accompanied by reduced generation of the 180 kDa FN cleavage fragment produced by ADAMTS4 proteolysis [40]. No adverse effects were observed with 13n treatment throughout the study [40]. Clinical study conducted by Vojtusek et al. suggests that ADAMTS4 may serve as a novel diagnostic biomarker for renal fibrosis [134]. It is supported by its significantly elevated immunohistochemical expression in the peritubular capillaries and interstitial spaces of patients with chronic kidney disease compared to controls, along with stepwise increases in abundance correlating with the progression of interstitial fibrosis [134]. The plasma analysis also confirmed the presence of circulating ADAMTS4 in patients with chronic kidney disease, peaking in stages 2 and 3, with subsequent disappearance by stage 5, which can fit into the dynamics of the development of the disease [134]. Additionally, Western blot and ELISA analyses revealed significantly elevated ADAMTS4 protein levels in serum samples from patients with myocardial infarction and dilated cardiomyopathy compared to controls, indicating its potential as a novel biomarker for cardiac injury in adults [135].

The endogenous regulator extracellular matrix protein 1 (ECM1) attenuates liver fibrosis in mice by targeting ADAMTS1 [131]. Specifically, ECM1 interacts directly with the KTFR motif within ADAMTS1, sequences known to mediate binding to the LAP and facilitate release of active TGF-β [131]. ECM1 inhibits the interaction between ADAMTS1 and LAP, downregulating TGF-β signaling [131]. Similarly, the exogenous agent LSKL peptide ameliorates unilateral ureteral obstruction induced renal fibrosis in mice by targeting the KPFR sequence in ADAMTS18, which is responsible for its interaction with LAP [90]. Witsch et al. reported that recombinant human ADAMTS13 (rhADAMTS13) ameliorates coronary microvascular dysfunction and improves cardiac remodeling in mice following left ventricular pressure overload [88]. Recently, clinical trials employing recombinant ADAMTS13 for the treatment of thrombotic thrombocytopenic purpura have demonstrated promising outcomes, suggesting its potential repurposing as a novel therapeutic agent against fibrotic cardiac injury [136].

Notably, endocytosis serves as a crucial post-translational regulatory mechanism for certain ADAMTSs, profoundly influencing their extracellular levels and often overriding transcriptional control [137]. It has been demonstrated that LRP-1 mediated endocytosis of ADAMTS4 and ADAMTS5 suppresses their capacity to degrade aggrecan within the ECM [138]. Specifically, ADAMTS4 and ADAMTS5 are internalized and cleared by chondrocytes via binding to LRP-1 through their non-catalytic domains. This endocytic system is impaired in osteoarthritic, which is characterized by excessive degradation of the ECM in articular cartilage. Research has shown that in osteoarthritic, increased ectodomain shedding of LRP-1 by the membrane-bound metalloproteinases ADAM17 and MMP14 releases soluble LRP-1, which binds to ADAMTS5 and blocks its clearance, thereby accelerating cartilage matrix degradation [139]. Besides, ADAMTS9 and ADAMTS20 have been identified to be internalized by LRP-1 and LRP-2 mediated endocytosis, a process crucial for ciliary vesicle growth during ciliogenesis [140]. These findings collectively suggest that LRP mediated endocytosis may represent a common regulatory mechanism for ADAMTS proteases. This regulatory paradigm offers a dual-path therapeutic strategy for fibrosis. On one hand, for pro-fibrotic ADAMTS members, enhancing their endocytic clearance could reduce their pathogenic extracellular activity. This could be achieved, for example, by inhibiting the pathological shedding of LRP--mediated by ADAM17 and MMP14, a mechanism demonstrated in osteoarthritis to restore protease clearance and attenuate matrix degradation [139]. On the other hand, for protective ADAMTS proteases such as ADAMTS18, strategies that block their internalization may prolong their residence in the extracellular milieu and amplify their beneficial functions. The feasibility of this approach is exemplified by a monoclonal antibody that specifically inhibits ADAMTS5 binding to LRP-1, thereby increasing its extracellular retention and activity without altering its catalytic function [141]. Consequently, targeting the LRP mediated endocytic regulation of ADAMTS proteases holds significant promise as a novel therapeutic strategy for fibrotic diseases. Additional endogenous and exogenous agents capable of modulating ADAMTS activity have been documented. Rose et al. provided a summary of these regulators [142]. This underscores the feasibility and prospects of developing more ADAMTS-targeted therapies for fibrosis treatment.

Despite several preclinical evidence highlighting ADAMTS proteases as promising therapeutic targets for fibrotic diseases, their translational progress toward clinical application has been limited. This disparity stems from several interconnected biological and pharmacological challenges. A fundamental obstacle is the context-dependent functional duality of individual ADAMTS members, where the same protease may exert opposing effects on fibrogenesis depending on the tissue microenvironment, disease stage, or specific substrate availability. A representative example is ADAMTS16: it can promote fibrogenesis by interacting with LAP-TGF-β via its RRFR motif to activate TGF-β signaling in cardiac and renal fibrosis, while concurrently exerting anti-fibrotic effects through the proteolytic cleavage of FN, which disrupts fibroblast adhesion, FAK phosphorylation, and collagen assembly in bicuspid aortic valve [6, 89, 108]. Besides, ADAMTS18 exhibits complex, context-influenced roles even within renal fibrosis: it can directly activate latent TGF-β to drive fibrosis through its KPFR-LAP interaction, yet it also cleaves FN, an action associated with the inhibition of fibroblast adhesion and FAK signaling, suggesting potential anti-fibrotic regulatory functions [90, 94]. This functional nuance highlights the challenge of predicting the net effect of ADAMTS modulation in disease. Further complicating therapeutic development is the substantial substrate overlap and functional redundancy within the ADAMTS family, where multiple proteases can process common substrates such as LAP and FN [7, 77, 89, 99, 108]. Consequently, inhibiting a single protease may yield insufficient efficacy due to compensatory mechanisms, while broader inhibition risks amplifying adverse effects by interfering with diverse physiological processes. The development of highly specific inhibitors remains technically challenging due to shared structural domains and catalytic mechanisms among the ADAMTS family members. Additionally, the field lacks a precise understanding of the spatiotemporal dynamics of ADAMTSs expression and activation throughout fibrotic progression, making it difficult to identify the optimal therapeutic window for intervention. These collective challenges, including functional pleiotropy, redundancy, limited drug specificity, and insufficient knowledge of temporal regulation, must be adequately addressed before ADAMTS-targeted therapies can advance toward clinical application.

## Conclusion

ADAMTS proteases critically regulate fibrosis by cleaving key ECM components such as collagens, FN, and proteoglycans, thereby modulating TGF-β activation, ECM stiffness, and fibroblast plasticity. Their functional impact is shaped by both context-dependent dual roles and extensive substrate redundancy within the family, which complicates therapeutic targeting due to compensatory mechanisms. Despite these challenges, promising preclinical approaches using specific inhibitors and biologics highlight their potential as therapeutic targets. Future efforts must unravel the spatiotemporal specificity and member-selective functions of ADAMTS proteases to enable the development of effective anti-fibrotic therapies.

## Data Availability

No datasets were generated or analysed during the current study.

## Abbreviations

ADAMTS: A disintegrin and metalloproteinase with thrombospondin motifs
ADAM: A disintegrin and metalloproteinase
MMP: Matrix metalloproteinase
ECM: Extracellular matrix
TSR: Thrombospondin type-1 repeat
TGF-β: Transforming growth factor-β
COMP: Cartilage oligomeric matrix protein
BMP: Bone morphogenetic protein
LOX: Lysyl oxidase
FN: Fibronectin
EDA: Extradomain A
EDB: Extradomain B
ATOMS: Amino terminal oriented mass spectrometry of substrate
TMT: Tandem mass tag
GAG: Glycosaminoglycan
LC-MS/MS: Liquid chromatography-tandem mass spectrometry
FBN: Fibrillin
SLRP: Small leucine-rich repeat proteoglycan
CCl₄: Carbon tetrachloride
vWF: von Willebrand factor
TGF-βRⅢ: TGF-β receptor III
FAK: Focal adhesion kinase
HMCN1: Hemicentin 1
JAK: Janus kinase
STAT: Signal transducer and activator of transcription
ACEi: Angiotensin converting enzyme inhibitor
TIMP-1: Tissue inhibitor of metalloproteinases-1
MMP-9: Matrix metalloproteinase-9
PPS: Pentosan polysulfate
ECM1: Extracellular matrix protein 1
rhADAMTS13: Recombinant human ADAMTS13
OPN: Osteopontin
LRP: Low-density lipoprotein receptor-related protein
